# Diverse Chemistry of Stable Hydronitrogens, and Implications for Planetary and Materials Sciences

**DOI:** 10.1038/srep25947

**Published:** 2016-05-19

**Authors:** Guang-Rui Qian, Haiyang Niu, Chao-Hao Hu, Artem R. Oganov, Qingfeng Zeng, Huai-Ying Zhou

**Affiliations:** 1 Department of Geosciences, Center for Materials by Design, and Institute for Advanced Computational Science, State University of New York, Stony Brook, NY 11794-2100, USA; 2 Guangxi Key Laboratory of Information Materials, Guilin University of Electronic Technology, Guilin 541004, P.R. China; 3 School of Materials Science and Engineering, Guilin University of Electronic Technology, Guilin 541004, P.R. China; 4 Skolkovo Institute of Science and Technology, Skolkovo Innovation Center, 3 Nobel St., Moscow 143026, Russia; 5 Moscow Institute of Physics and Technology, 9 Institutskiy lane, Dolgoprudny city, Moscow Region 141700, Russia; 6 International Center for Materials Discovery, School of Materials Science and Engineering, Northwestern Polytechnical University, Xi’an 710072, P.R. China

## Abstract

Nitrogen hydrides, e.g., ammonia (NH_3_), hydrazine (N_2_H_4_) and hydrazoic acid (HN_3_), are compounds of great fundamental and applied importance. Their high-pressure behavior is important because of their abundance in giant planets and because of the hopes of discovering high-energy-density materials. Here, we have performed a systematic investigation on the structural stability of N-H system in a pressure range up to 800 GPa through evolutionary structure prediction. Surprisingly, we found that high pressure stabilizes a series of previously unreported compounds with peculiar structural and electronic properties, such as the N_4_H, N_3_H, N_2_H and NH phases composed of nitrogen backbones, the N_9_H_4_ phase containing two-dimensional metallic nitrogen planes and novel N_8_H, NH_2_, N_3_H_7_, NH_4_ and NH_5_ molecular phases. Another surprise is that NH_3_ becomes thermodynamically unstable above ~460 GPa. We found that high-pressure chemistry of hydronitrogens is much more diverse than hydrocarbon chemistry at normal conditions, leading to expectations that N-H-O and N-H-O-S systems under pressure are likely to possess richer chemistry than the known organic chemistry. This, in turn, opens a possibility of nitrogen-based life at high pressure. The predicted phase diagram of the N-H system also provides a reference for synthesis of high-energy-density materials.

Hydrogen is the most abundant, and nitrogen is the seventh most abundant element in the universe. Giant planets Uranus and Neptune are predominantly made of H, O, C and N. While the behavior of the H-O^1^ and C-O^2^ systems under pressure has been investigated in some detail, the N-H system remains largely unexplored. Ammonia (NH_3_), an important compound in many branches of science and technology, was first proposed to exist in Uranus and Neptune by Ramsey^3^ and Bernal and Mussey^4^ in early 1950 s, and further discussed by Stevenson and Bundy^5^,^6^. It is the only stable hydronitrogen at ambient conditions, and exists in a wide range of temperatures and pressures. Recent studies^7^,^8^,^9^ revealed that ammonia undergoes a series of phase transitions, including ionic disproportionation and return to non-ionic phase at megabar pressures. Ammonia is considered as a major component of the interiors of giant planets such as Uranus and Neptune under extreme pressure (up to 600 GPa) and temperature (2,000∼7,000 K)^10^,^11^,^12^,^13^,^14^. What has not been properly explored is the full phase stability in the N-H system, including the possibility of decomposition of ammonia; it may well be that, instead of ammonia, very different molecules with different stoichiometries are actually present in planetary interiors.

All nitrogen hydrides, except ammonia, are metastable at ambient pressure. Due to the substantial energy difference between single and triple nitrogen-nitrogen bonds, nitrogen-rich hydronitrogens are potentially superior high-energy-density materials. However, large-scale synthesis of these materials is still problematic. Having a complete phase diagram for the N-H system is necessary for developing synthetic strategies, but such a phase diagram has not been determined. As a result, the high-pressure behavior, structures and stability of N-H phases are of great interest to both planetary and condensed-matter physics.

Extensive theoretical^15^,^16^,^17^,^18^ and experimental^19^ studies revealed exotic compounds appearing under compression, and exhibiting unique structures and properties different from usual compounds -see previous investigations of Na-Cl^19^, Mg-O^17^, B-H^16^, H-O^1^ and Mg-Si-O^18^ systems. Considering the dramatically changed nature of nitrogen^20^,^21^,^22^ and the autoionization^7^ found in NH_3_, new hydronitrogen compounds are expected to be found.

## Results

### Stoichiometries and structures

**Figure 1:**
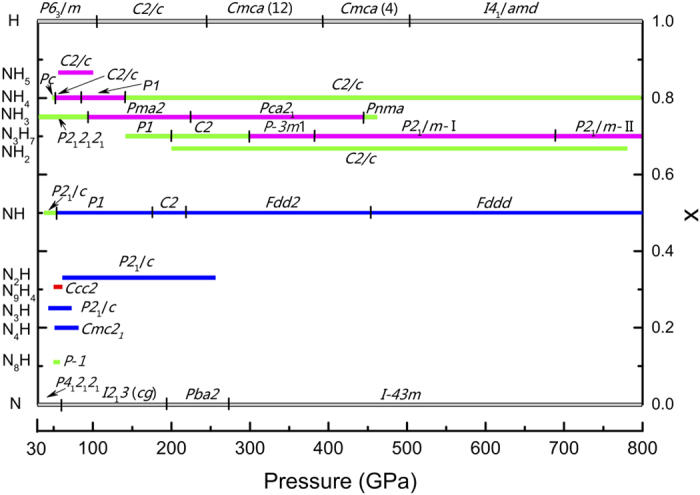
Phase diagram for N-H system from 30-800 GPa. For hydronitrogen phases blue color indicates infinite nitrogen chain structures. Green and pink molecular and molecular ionic structures, respectively. The red color indicates the 2D-polymeric N_9_H_4_ phase.

**Figure 2:**
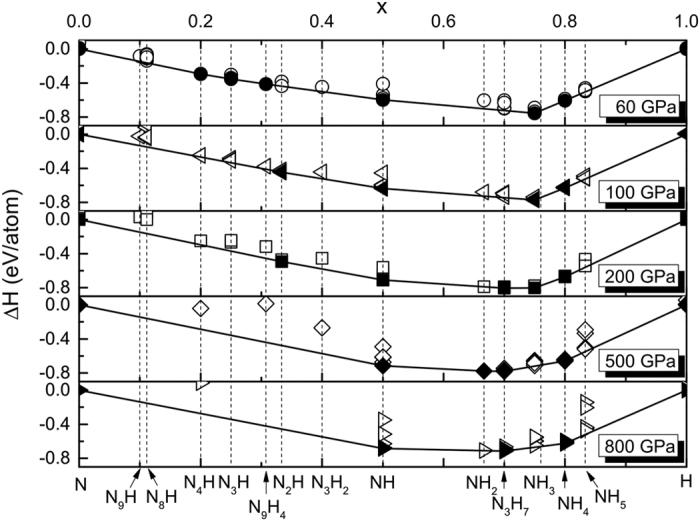
Convex hull for the N-H system at 60, 100, 200, 500, 800 GPa. The full and open symbols indicate stable and metastable phases, respectively.

Using the evolutionary algorithm USPEX^23^,^24^,^25^,^26^, we have carried out structure and stoichiometry predictions in order to find all stable compounds (and their stability fields) in the N-H system (See Methods). Our calculations confirm that ammonia is the only stable hydronitrogen from ambient pressure to 36 GPa. Above 36 GPa, remarkably, a series of previously unknown compounds become stable, as shown in the pressure-composition phase diagram of the N-H system in Fig. 1. The detailed convex hulls at 60, 100, 200, 500, and 800 GPa are presented in Fig. 2. It needs to be emphasized that by calculating phonon dispersions, all the newly found compounds in this work are found to be dynamically stable in their corresponding stability field on the phase diagram (Fig. 1). Since zero-point energy can be a factor to affect the relative stability of structures, we have done zero-point energy calculations for N_2_H, NH, N_3_H_7_, NH_4_ and NH_7_, and found out that the phase diagram shown in Fig. 1 does not change significantly. Therefore, in this work, the phase diagram of N-H is drawn without considering zero-point energy. We would like to leave more accurate phase diagram investigation of N-H at finite temperatures and with considering zero-point energy for further work.

We can classify these thermodynamically stable hydronitrogens compounds that we found into three types (See Table 1). (i) Infinite-chain polymeric hydronitrogens, including N_4_H, N_3_H, N_2_H and NH, with polymeric chains featuring all-nitrogen backbones. (ii) Two-dimensional (2D) metallic N_9_H_4_ phase consisting of 2D nitrogen planes and NH^+^_4_ cations, interestingly, the 2D-nitrogen planes have not been reported in any other nitrogenous compounds before. (iii) Molecular compounds including N_8_H, NH_2_,N_3_H_7_, NH_4_, NH_5_, and of course NH_3_. Here, molecular (or molecular ionic) compounds are bonded by hydrogen bonds.

### One-dimensional polymeric hydronitrogens

**Figure 3:**
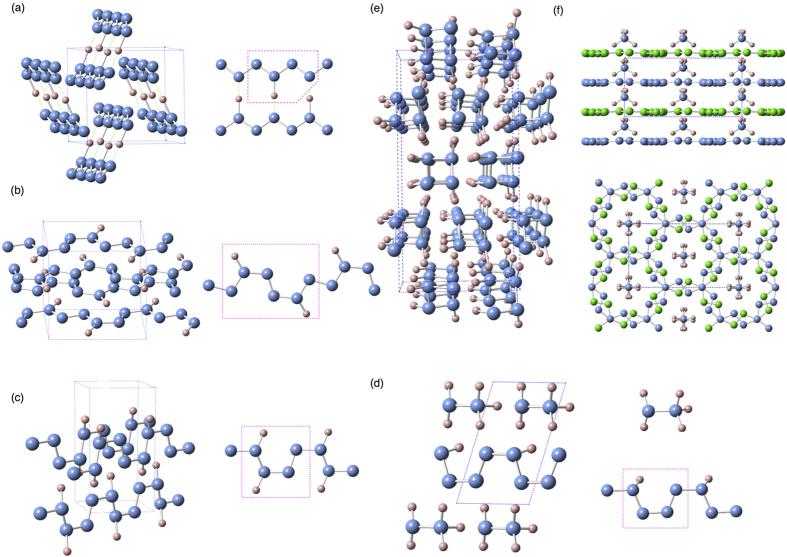
The proposed structures of N_4_H, N_3_H, and N_2_H and NH, N_9_H_4_. The small pink spheres indicate hydrogen atoms and the blue large spheres are nitrogen atoms. (**a**) *Cmc*2_1_-N_4_H structure. The structure is composed of one-dimensional zigzag-shaped N-chains. Every two chains are engaged though asymmetric hydrogen bonds. (**b**) Layered *P*2_1_/*c*-N_3_H structure containing distorted arm-chair-shaped chain. (**c**) *P*2_1_/c-N_2_H structure composed of parallel one-dimensional arm-chair-shaped N_2_H chains. (**d**) *P*1-NH structure. Its structure consists of N_2_H_5_^+^ ions and negatively charged arm-chair-shaped chain layers. It will transform to *C*2 phase at 180 GPa, due to the symmetrization of the hydrogen bonds between N_2_H_5_^+^ ions and between chains. (**e**) The *Fdd*2-NH structure consists of pseudotetragonal spiral chains. (**f**) Top view and side view of *Ccc*2-N_9_H_4_. The small pink spheres indicate hydrogen atoms and the blue and green large spheres are nitrogen atoms at different layers.

We have found that, except N_9_H_4_ and N_8_H, nitrogen-rich hydronitrogens (N_x_H, x ≥ 1) are more prone to adopt polymeric structures with N-backbones, The N_4_H, N_3_H and N_2_H compounds are predicted to be stable at 51–80 GPa, 42–75 GPa and 60–260 GPa, respectively. The ground state of N_4_H has a *Cmc*2_1_ structure, containing four zigzag nitrogen chains (N-chains) in the unit cell, with pairs of nearest N-chains linked by hydrogen bonds, see Fig. 3(a). Here, we use [
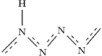
] to represent the monomeric unit in the polymeric chain of N_4_H. The delocalized nitrogen-nitrogen bonds run along the zigzag chain, and have the same length of 1.28 Å at 60 GPa. Instead of a zigzag chain, the most stable N_3_H structure has space group *P*2_1_/*c* and is composed of distorted arm-chair monomers [
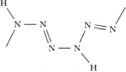
], see Fig. 3(b). These chains are connected with each other through H-bonds to form a layered structure. The *P*2_1_/*c* phase of N_2_H becomes thermodynamically stable at ~60 GPa, and its structure consists of two [
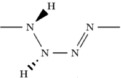
] monomers in the unit cell, see Fig. 3(c). At 200 GPa, the lengths of single N-N bonds in this polymer are 1.27 and 1.28 Å, and the double N=N bond is slightly shorter (1.24 Å). The smallness of the difference hints at a possible bond resonance along the chain. The doubly-bonded nitrogen atoms form weak asymmetric hydrogen bonds with nearby chains. Before the symmetrization of hydrogen bonds occurring at ∼280 GPa, *P*2_1_/*c* -N_2_H undergoes a spontaneous decomposition at ∼260 GPa. All these polymeric structures are metallic as a result of bond resonance and electronic delocalization along the nitrogen backbone.

With the equal ratio of nitrogen and hydrogen, the NH compound is predicted to be stable in a huge pressure range, from 36 GPa to at least 800 GPa. The *P*2_1_/*c* structure is more stable than the one predicted in the work of Hu & Zhang^27^. This phase consists of two tetrazene N_4_H_4_ molecules in the unit cell. At 55 GPa, *P*2_1_/*c* -NH undergoes a phase transition to an ionic structure with *P*1 symmetry. As shown in Fig. 3(d), the ionic structure is composed of N_2_H_5_^+^ cations arranged in hydrogen-bonded layers, alternating with layers of infinite chains [
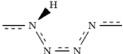
]^−^. The unit cell contains 6 NH formula units: N_2_H_5_^+^ group and N_4_H^−^ from the polymeric chain. At ∼180 GPa, all hydrogen bonds become symmetric and the space group raises to *C*2. Both *P*1 and *C*2 are only nominally ionic, because they are metallic and metals have very efficient screening of ionic interactions by the electron gas. Above ∼220 GPa, the ionic NH phases become less stable than an *Fdd*2 structure which is made of tetragonal spiral chains, as shown in Fig. 3(e). Similar square chains have been reported in group VI elements under pressure, e.g. sulfur-II phase^28^ and the *I*4_1_/*amd* phase of oxygen at pressure around 2 TPa^29^. The *Fdd*2-NH is predicted to be a wide-gap semiconductor (4.8 eV at 400 GPa). In contrast to the strongly localized electrons found in the *I*4_1_/*amd*-oxygen structure with isolated chains, *Fdd*2-NH has asymmetric hydrogen bonds between the square chains. *Fdd*2 transforms to an *Fddd* structure upon hydrogen bond symmetrization at 460 GPa. For both these orthorhombic phases symmetry breaking leads to two non-equivalent N-N bond lengths in the chain -e.g. 1.25 and 1.34 Å in *Fddd*-NH at 460 GPa. The different lengths of the N-N bonds come from the distortion of the square spirals, caused by their packing and hydrogen bond pattern. The *Fddd*-NH remains stable up to at least 800 GPa.

### 2D-polymeric hydronitrogen

Distinct from the polymeric chain structures, we also discovered an exotic stable nitrogen-rich compound N_9_H_4_. Its structure has *Ccc*2 symmetry, and is composed of negatively-charged 2D nitrogen planes and NH_4_^+^ cations. *Ccc*2-N_9_H_4_ was predicted to be thermodynamically stable in a narrow pressure range 50–60 GPa. As shown in Fig. 3(f), the 2D nitrogen plane is a loose structure due to the hexagonal star-shaped holes decorated by 18 additional nitrogen atoms. Parallel stacking of the nitrogen planes creates infinite channels in the perpendicular direction, and NH_4_^+^ cations are located along these channels. The electrons in the plane are delocalized, as a result this compound is metallic with a flat band crossing the Fermi level. (See more details about properties of N_9_H_4_ in Supplementary Information).

### Molecular hydronitrogens

N_8_H is found to be stable around 50 GPa, and adopts a very unusual molecular structure with four pentazole (N_5_H) and six nitrogen (N_2_) molecules in the unit cell. (See more details about N_8_H structure in Supplementary Information).

**Figure 4:**
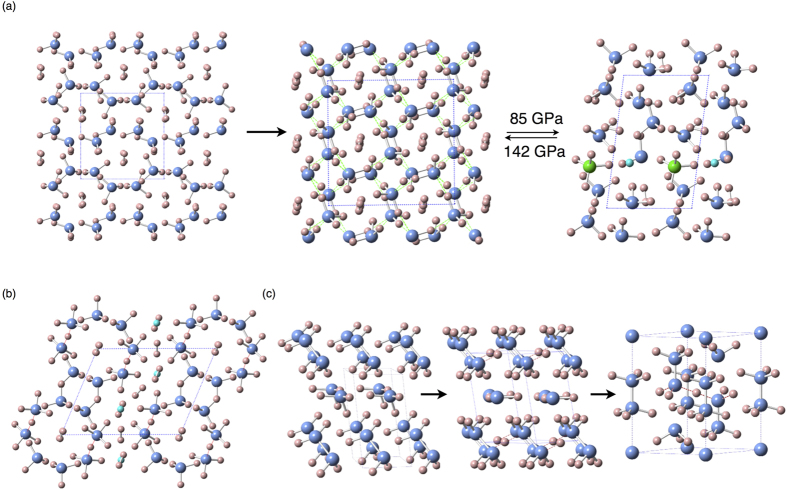
The proposed structures of NH_4_, NH_5_ and N_3_H_7_. The small pink spheres indicate hydrogen atoms and the blue large spheres are nitrogen atoms. The nitrogen atom in NH_4_^+^ cation and the H^−^ anion are noted with green and aqua spheres, respectively. (**a**) Phase transition sequence from host-guest *Pc*→host-guest *C*2/*c*↔Partially ionic *P*1-NH_4_ phases. In host-guest structure of *C*2/*c*-NH_4_, the hydrogen molecules are captured in the channels formed by NH_3_ molecules. In the partially ionic *P*1-NH_4_ structure, the NH_4_^+^ cation is close to the H^−^ anion. (**b**) The ionic *C*2/*c* NH_5_ phase, with symmetric hydrogen bonds in [H_3_N···H···NH_3_]^+^ units and H^−^ anions. (**c**) Phase transition sequence molecular *P*1→ionic *C*2→ionic *P*-3*m*1 N_3_H_7_.

Hydrogen-rich hydronitrogens, instead of polymeric structures, have hydrogen-bonded molecular structures. The NH_4_ phases, containing a higher hydrogen ratio than NH_3_, are found to be thermodynamically stable above ∼50 GPa, and remain stable at least up to 800 GPa. At pressures above 50 GPa, NH_4_ first adopts a host-guest structure of *Pc* symmetry with the structural formula (NH_3_)_2_·H_2_. Other host-guest structures, adopting *P*2_1_, *C*2/*c* and *I*4/*m* symmetries, have very close enthalpies to this structure below 80 GPa (See more details about these NH_4_ structures in Supplementary Information). Accurate fixed-composition crystal structure predictions for NH_4_ show that above 52 GPa, *C*2/*c* structure is located in the global minimum of the energy landscape for NH_4_, and other structures with close enthalpies are structurally similar to the *C*2/*c* structure. In all host-guest structures, H_2_ molecules are captured in hydrogen-bonded frameworks formed by NH_3_ molecules. In the pressure range 85–142 GPa, the ionic *P*1-NH_4_ phase is more stable than host-guest molecular structures. In the unit cell of this low-symmetry ionic phase, as shown in Fig. 4(a), every eighths ammonia molecule reacts with an H_2_ molecule to form the NH_4_^+^ cation and H^−^ anion. The distance of H^−^ anion and the nearest hydrogen of the NH^+^_4_ cation is 1.13 Å at 100 GPa. Above 142 GPa, the ionic phase undergoes a reentrant transition to the same *C*2/*c* host-guest structure again, thus returning to structures consisting of neutral NH_3_ and H_2_ molecules. Hydrogen-bond symmetrization was not observed in all stable NH_4_ phases up to 800 GPa.

With the 1:1 ratio of H_2_ and NH_3_, several NH_5_ phases are also found to be thermodynamically stable or nearly stable around 55–100 GPa. The ionic *C*2/*c* phase (See Fig. 4(b)) has the lowest enthalpy at pressures below 162 GPa. In the unit cell of *C*2/*c*-NH_5_, there are two [H_3_N···H···NH_3_]^+^ units and two H^−^ anions. At pressure above ~162 GPa, *C*2/c-NH_5_ phase transforms into metastable ionic *P*2 and *Ama*2 structures, then adopts a *P*2_1_/*c* structure containing alternating layers of NH_3_ and H_2_ molecules above ∼363 GPa. (See more details about NH_5_ high pressure phases in Supplementary Information).

At about 140 GPa, a previously unreported remarkable compound with the composition N_3_H_7_ is also found to be thermodynamically stable. For N_3_H_7_, we have predicted several thermodynamically stable phases with the structural sequence *P*1 → *C*2 → *P*-3*m*1 → *P*2_1_/*m*-I → *P*2_1_/*m*-II upon increasing pressure (See Fig. 4(c) for the first three structures). At 140–200 GPa, *P*1-N_3_H_7_ adopts a stable molecular structure, consisting of one ammonia (NH_3_) and one hydrazine (N_2_H_4_) molecules in the unit cell. At 200 GPa, *P*1 undergoes a spontaneous molecular-to-ionic transition, resulting in a layered *C*2 structure. In this process, ammonia and hydrazine molecules react to form the NH_2_^−^ (amide) anions and N_2_H^+^ (hydrazinium) cations, respectively. The N_2_H^+^ ions are in a parallel arrangement and connected by symmetric H-bonds. At 300–380 GPa, complicated ionic N_3_H_7_ structure of *P*-3*m*1 symmetry becomes stable. As shown in Fig. 4(c), in this unique structure, the trigonal unit cell has two neutral ammonia molecules, one N^3−^ anion, one [N_2_H_6_]^2+^ cation and one [
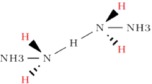
]^+^ unit (net formula N_4_H_9_^+^, the red H symbols indicate that such hydrogen atoms are symmetrically hydrogen-bonded and shared with neighbor N_4_H_9_^+^ units). This is the only structure with bare nitrogen anions observed among the newly proposed nitrogen hydrides. The nitride anion N^3−^ is surrounded by 12 hydrogen atoms from NH_3_ molecules and N_4_H_9_^+^ cation, with distances of 1.32 and 1.38 Å at 380 GPa. Then, at pressure above 380 GPa, the trigonal N_3_H_7_ phase will give way to another two *P*2_1_/*m* type ionic structures, consisting of NH_2_^−^ anions and N_2_H_5_^+^ cations again. They have different packing patterns from the ionic *C*2 structure (named *P*2_1_/*m*-I and *P*2_1_/*m*-II N_3_H_7_ by stability sequence upon increasing pressure, respectively (See Supplementary Information for more details).

With pressure increasing, our calculation confirmed that NH_3_, above 36 GPa, undergoes phase transformations from hydrogen-bonded molecular *P*2_1_2_1_2_1_ structure to layered ionic *Pma*2 and *Pca*2_1_ phases, and then returns to *Pnma* structures consisting of neutral NH_3_ molecules at very high pressure^7^,^9^. However, NH_3_, the only thermodynamically stable hydronitrogen compound at ambient conditions, is surprisingly predicted to decompose into N_3_H_7_ and NH_4_ at ~460 GPa at zero temperature. For NH_2_, the dense molecular hydrazine phase was also predicted to be stable and have a *C*2/*c* symmetry at ~200–780 GPa, which is consistent with Zhang’s work^30^. The *C*2/*c* structure of NH_2_ consists of hydrazine molecules, forming both symmetric and asymmetric hydrogen bonds with each other.

## Discussion

Our theoretical calculations indicate that the N-H system exhibits rich chemistry under pressure. The infinite long-chain polymeric structures are widely found in nitrogen-rich hydronitrogen compounds, and are thermodynamically stable above 42 GPa. They could potentially serve as good high-energy-density and fuel materials due to the substantial energy difference between the single/double and triple nitrogen-nitrogen bonds. The nitrogen backbone evolves with increasing hydrogen concentration. With the “antiseeds” technique (See Methods), we found that metastable nitrogen phases containing zigzag N-chains have competitive enthalpies (~0.03 eV/atom higher at 60 GPa) to the molecular states and the singly bonded cg-N^31^ structure at 40–70 GPa, and they are more energetically favorable than arm-chair-shaped and other N-chains (See Fig. S5 in Supplementary Information). A low hydrogen content stabilizes these chains and does not change much of the packing pattern of the chains and the electronic properties of the resonant N-N bonds. The lowest-enthalpy structures of metastable N_9_H and stable N_8_H phases contain infinite zigzag N-backbones. (See more details about these two compounds in Supplementary Information) With higher hydrogen content, the zigzag N-backbone become unstable in N_3_H, N_2_H and NH phases.

**Figure 5:**
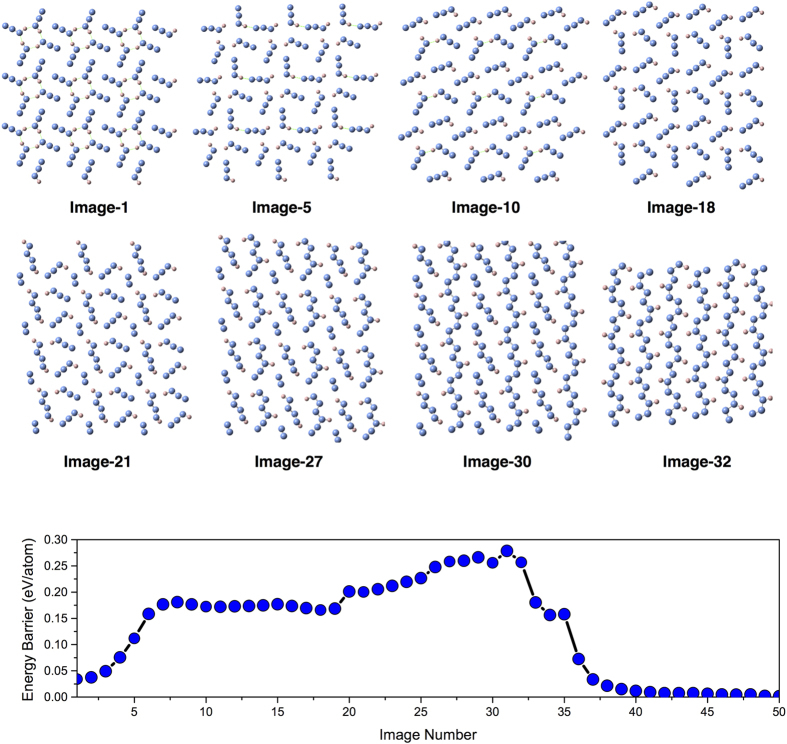
Mechanism of polymerization of hydrazoic acid (from molecular to polymeric *P*2_1_/*c* N_3_H) revealed by the VC-NEB method. A unit cell with 32 atoms was used during the pathway calculation. Only one layer of N_3_H structures during the phase transition is shown at specific images.

These long-chain polymeric hydronitrogen compounds would be an interesting alternative to commonly used high-energy-density materials. Compared to pure polymeric nitrogen (cg-N phase), layered *P*2_1_/*c* N_3_H is stable at pressures above ~42 GPa, i.e. at pressures lower than the stability pressure of cg-N (>56 GPa). Hydrazoic acid^32^ (N_3_H) may be an even better precursor for synthesizing long-chain polymers. With hydrazoic acid, the layered *P*2_1_/*c* N_3_H can be formed at as low as 6.0 GPa (See Table 2). The VC-NEB^33^ calculation indicates that the phase transformation from hydrazoic acid to *P*2_1_/*c* N_3_H has an energy barrier of ~0.25 eV/atom at 10 GPa, (See Fig. 5), and occurs in several stages. In the first stage, some H-bonds between HN_3_ molecules break, making the molecules free to rotate (as shown in Fig. 5 from Image-1 to Image-5). After adjusting directions of HN_3_ molecules (Image-5 to Image-18), metastable short N-chain molecules (Image-21 and Image-27) appear during the transition, new nitrogen-nitrogen bonds appear, eventually leading to infinite polymeric chains (Image-30). The energy barrier of first stage with rotation of the HN_3_ molecules is around 0.15 eV (from Image-1 to Image-19), and approximately equals to the barrier of the second stage (nitrogen-nitrogen bond formation). The transition should happen easily in liquid hydrazoic acid. Mixture of hydrazine and hydrazoic acid is an alternative precursor, with polymerization estimated to happen at ∼13 GPa (See Table 2).

Isoelectronic to oxygen, (NH) units generally serve as analogs of group VI elements in these polymeric chain structures. Besides the square-spiral chain in high-pressure phases found in NH, the monoclinic N_2_H phase can be considered as an analogue material of sulfur nitride (SN)_n_^34^ or (ON)_n_^35^,^36^ polymers. The proposed nitrogen oxides (ONNO)_n_ chain oligomer also has comparatively strong N=N bonds. The monoclinic N_2_H phase is a metallic polymer as the Fermi level is crossed by anti-bonding π* bands (See Fig. S4 in Supplementary Information), which is similar to the first known metallic polymer (SN)_n_^37^ as a superconductor with T_*c*_ = 0.26 K^38^. All our 1D long-chain hydronitrogen compounds containing delocalized nitrogen bonds are metallic. Our calculations reveal that N_4_H (at 55 GPa) and N_2_H (at 60 GPa) are superconductors with T_*c*_ = 2.6 and 7.8 K (with the value of μ* = 0.13), respectively. In contrast, N_9_H_4_ phase is not a superconductor.

Multiple stable stoichiometries exist in hydrogen-rich hydronitrogens at pressure. These hydronitrogens form molecular crystals at low pressure, and then tend to undergo auto-ionization under moderate compression, except NH_2_ (See Table 1). The structures of these compounds show various characteristics and are quite different from each other. N_3_H_7_, NH_4_ (and NH_5_) can be considered as binary NH_3_ + N_2_H_4_ and NH_3_ + xH_2_ compounds, respectively. Therefore, in general, high-pressure hydrogen-rich hydronitrogens tend to contain molecules and molecular ions.

It is predicted that hydrogen-rich hydronitrogens remains stable to extremely high pressures, NH_3_ and NH_2_ become unstable and decompose (into NH_4_ and N_3_H_7_, or into NH and N_3_H_7_) only at 480 and 780 GPa, respectively; and NH_4_ and N_3_H_7_ are thermodynamically stable at least up to 800 GPa. In contrast, methane (CH_4_) was predicted to dissociate into ethane (C_2_H_6_), butane (C_4_H_10_), and finally, diamond plus hydrogen at 287 GPa^2^.

NH_4_ and NH_5_ undergo a molecular⇒ionic⇒molecular phase sequence under pressure, which is very similar to NH_3_^7^. The auto-ionization process also occurs in N_3_H_7_, which remains in the ionic phase at least up to 800 GPa. In contrast, C-H compounds have non-polar non-ionic structures, and the high energy cost of proton transfer in H_2_O^7^,^39^ prevents auto-ionization until extremely high pressure (~1.4 TPa)^39^. Our calculation revealed that the energy cost of proton transfer from H_2_ to NH_3_ molecule and from NH_3_ to N_2_H_4_ molecule is ~0.7 eV and ~1.0 eV, respectively, while it costs ~0.9 eV^40^ to form NH^−^_2_ and NH^+^_4_ ions in NH_3_. Therefore, NH_3_ + xH_2_ compounds would undergo auto-ionization at a lower pressure (NH_4_ at ~85 GPa and NH_5_ at ~42z GPa) than pure NH_3_(at ~90 GPa). Due to high cost of proton transfer, auto-ionization phenomenon was not observed in any stable H_2_O-H_2_ compounds^1^. Calculations show that auto-ionization happens at ~200 GPa in N_3_H_7_, higher in NH_3_ (90 GPa)^7^, due to the higher proton transfer energy cost, and survives up to at least 800 GPa. The pV term in the free energy plays an important role in deterring the phase transition sequence at high pressure. Under pressure, stable N_3_H_7_ and NH_3_ -xH_2_ host-guest phases are more packing-efficient than the volume of NH_3_ + N_2_H_4_ and NH_3_ + H_2_ mixtures, respectively. The auto-ionization transition in N_3_H_7_ leads to denser structures and enhances stability of N_3_H_7_ under compression.

## Conclusions

We have extensively explored the nature of hydronitrogen compounds up to ultrahigh pressures. It turns out that unusual compounds, such as N_8_H, N_4_H, N_3_H, N_9_H_4_,N_2_H, NH, NH_2_,N_3_H_7_, NH_4_ and NH_5_ are stable under pressure. These compounds possess intriguing crystal structures and remarkably novel, exotic properties. Three main features can be concluded, 1) the (NH) unit behaves similarly to its isoelectronic analogs, oxygen (also the sulfur) atoms, 2) molecular hydronitrogens are mainly composed of H_2_, NH_3_,N_2_H_4_ molecules and corresponding ions, 3) auto-ionization is common in N-H molecular phases due to the low energy cost of the proton transfer between the H_2_, NH_3_,N_2_H_4_ molecules.

Our investigation opens ways for designing synthesis of novel high-energy-density polymeric hydronitrogens. It is clear that starting with metastable precursors (such as N_2_H_4_, N_3_H) should lower polymerization pressure (compared to the lowest pressure of thermal dynamic polymeration, 42 GPa). We experimented with different mixtures of N_2_H_4_, N_3_H and N_2_ give bulk N_3_H or NH compositions. We found that using N_2_ in the precursor mixture does not give good results. Instead, pure N_2_H_4_ and N_3_H, or their mixtures can polymerize already at near-ambient conditions. For planetary interiors (where H/N > 1), we expect the presence of N-contianing molecular ions at all pressures above ~55 GPa in NH_5_. This means a much thicker layer with ionic conductivity than previously thought, which will affect models of planetary magnetic fields (which are generated by convection of electrically conducting layers). High-pressure chemistry of hydronitrogens uncovered here has greater diversity than hydrocarbons.

We remind that at normal conditions, the only thermodynamically stable compound of carbon and hydrogen is methane (CH_4_), all the other hydrocarbons being metastable and kinetically protected by high energy barriers. Here we have uncovered unique structural diversity among THERMODYNAMICALLY STABLE hydronitrogens. N-H bonds are directional covalent bonds (just like C-H), which should also lead to high energy barriers and ubiquitous metastability. If one includes metastable hydronitrogens, and adds other elements (such as O, S, smaller amounts of C), the diversity will most likely exceed the diversity of organic chemistry. This invites the question whether nitrogen-based (rather than carbon-based) life is possible in the interiors of gas giant planets. Briefly, we see the following conditions as necessary for emergence of life: (1) great structural and chemical diversity based on a small number of chemical elements (C-H-O or N-H-O), (2) abundance of metastable compounds with long lifetimes, (3) chemical reactions for energy production, (4) reversible reaction for storing/releasing energy (similar to the function of ATP in carbon-based life), (5) a molecule that can be used as information matrix (analogous in its function to DNA). For nitrogen-rich compounds, condition (1) is clearly satisfied. Condition (2) is also likely satisfied at not very high temperatures. Energy source and storage can be related to metastable compounds -e.g. oxidation of hydronitrogens for energy production, and polymerization/depolymerization of hydronitrogens for energy storage. As for condition (5), it is too early to say which N-based molecules could be suitable -the main conditions seem to be 1D-or 2D polymeric nature and aperiodicity. Nitrogen-based life could be possible, but the likelihood of this is highly limited due to high temperatures in these planets’ interiors, which could make lifetimes of metastable compounds too short. Given the abundance of N, H, O, C in giant planets, and high pressures in their interiors, we expect great diversity of molecular species there.

## Methods

### Crystal structure prediction

Crystal structure prediction was performed using the variable-composition evolutionary algorithm USPEX^15^,^23^,^24^,^25^,^26^. A number of studies illustrate the power of the USPEX method^16^,^17^,^19^. Calculations for the N-H system were performed at various pressures in the wide range of 0–800 GPa.

Given the dramatically changed behavior of nitrogen under pressure and a wide pressure range of our investigation, we performed a number of different types of predictions with USPEX. We ran variable-composition predictions for N-H, N-NH and NH-H systems with up to 32 atoms per unit cell. Given molecular nature of all stable and nearly stable compounds in hydrogen-rich hydronitrogens, we also did structure prediction for the packing of well-defined NH_3_ and H_2_ molecules (rather than N and H atoms), by applying the specially designed constrained global optimization algorithm^26^, considering structures with up to 24 molecules (i.e. up to 96 atoms) per primitive unit cell. These calculations were run together in a global coevolutionary search with exchanging good (stable and some metastable) structures between different runs. This coevolutionary method is very efficient and has been implemented on top of the USPEX code. When performing prediction for metastable nitrogen structures containing zigzag N-chains, we applied the antiseeds technique^41^, which was adopted to search for all low-enthalpy structures based on zigzag N-chains.

### DFT calculations

The underlying *ab initio* structural relaxations and electronic structure calculations in UPSEX were carried out using the all electron projector augmented wave (PAW)^42^ method as implemented in the VASP code^43^. The plane-wave cutoff energy of 800 eV and dense Gamma-centered k-point meshes with a resolution better than 2π×0.05 Å^-^ were adopted, and ensured high-quality results. After identifing the most stable compositions and several candidate structures, we relaxed them at numerous pressures in the range of 0–800 GPa with harder PAW potentials, in which the core radius equals 0.42 and 0.58 Å for hydrogen and nitrogen, respectively. An extremely high cutoff energy of 1400 eV was used for these relaxations and calculations of enthalpies of reactions and phase diagram. In addition, phonon dispersions throughout the Brillouin zone were derived using the finite-displacement approach as implemented in the Phonopy code^44^. Superconducting T_*c*_ was calculated in QUANTUM ESPRESSO^45^, with ultrasoft potentials^46^ using 40 Ry plane-wave cutoff energy.

## Additional Information

**How to cite this article**: Qian, G.-R. *et al*. Diverse Chemistry of Stable Hydronitrogens, and Implications for Planetary and Materials Sciences. *Sci. Rep.* **6**, 25947; doi: 10.1038/srep25947 (2016).

## Supplementary Material

Supplementary Information

## Figures and Tables

**Table 1:**
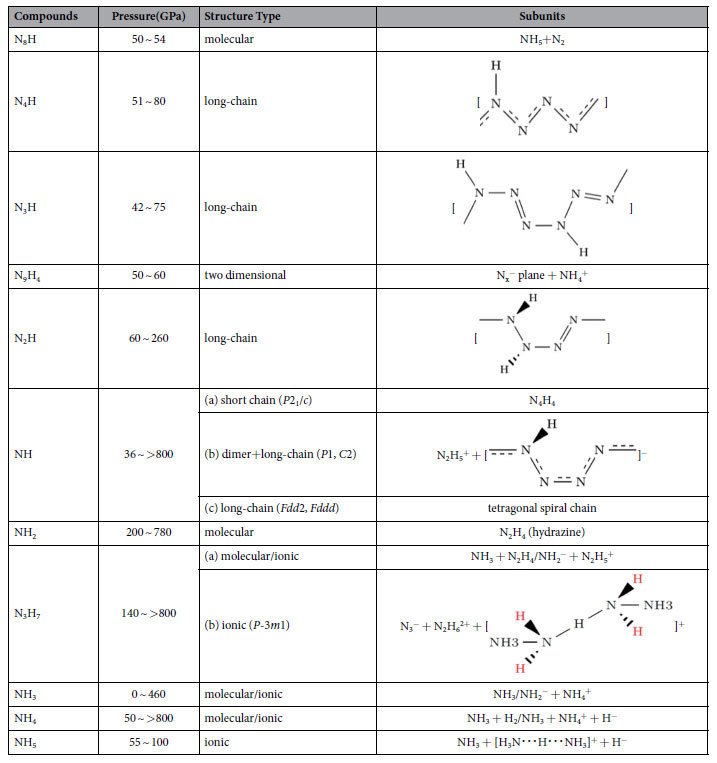
Chemical features of stable hydronitrogens.

**Table 2:** Chemical reactions to synthesis high-energy-density hydronitrogen at ΔH = 0.

	Reaction	Pressure [GPa]	ΔV
N_3_H (HA*)	→ N_3_H (long-chain)	6.0	7.58
N_2_H_4_ + N_3_H (HA)	→ 5NH (dimer+long-chain)	12.1, 12.8**	10.9, 9.81**
N_2_H_4_ + N_2_(*P*4_1_2_1_2_1_)	→ 4NH (dimer+long-chain)	32.5	6.26
N_2_H_4_ + 5N_2_(*P*4_1_2_1_2_1_)	→ 4N_3_H (long-chain)	37.3	18.5

^*^HA shorts for Hydrazoic Acid.

^**^With *C*2 and *P*2_1_-N_2_H_4_ phases, respectively.
