# Supplementary material for: Diverse Chemistry of Stable Hydronitrogens, and Implications for Planetary and Materials Sciences
Source: Sci Rep. 2016 May 19;6:25947. doi: 10.1038/srep25947 (PMC4872144; doi:10.1038/srep25947)
Supplement: Supplementary Information [file srep25947-s1.pdf]

Diverse Chemistry of Stable Hydronitrogens, and Implications for Planetary and Materials Sciences

Supplementary information

Guang-Rui Qian,[1] Haiyang Niu,[1] Chao-Hao Hu,[2,3] Artem R. Oganov,[4,5,1,6] Qingfeng Zeng,[6] and Huai-Ying Zhou[2]

[1]*Department of Geosciences, Center for Materials by Design, and Institute for Advanced Computational Science, State University of New York, Stony Brook, NY 11794-2100*

[2]*Guangxi Key Laboratory of Information Materials, Guilin University of Electronic Technology, Guilin 541004, P. R. China*

[3]*School of Materials Science and Engineering, Guilin University of Electronic Technology, Guilin 541004, P. R. China*

[4]*Skolkovo Institute of Science and Technology, Skolkovo Innovation Center, 3 Nobel St., Moscow 143026, Russia*

[5]*Moscow Institute of Physics and Technology, 9 Institutskiy lane, Dolgoprudny city, Moscow Region 141700, Russia*

[6]*School of Materials Science, Northwestern Polytechnical University, Xi'an 710072, China*

(Dated: February 15, 2016)

The N_8H phase is very close to the convex hull at 40∼60 GPa (as shown in Fig. S1), ∼0.003eV/atom above the convex hull line at 55 GPa. Below 69 GPa, the ground state of N_8H adopts the $P\bar{1}$-N_8H molecular structure with four pentazole (N_5H) and six nitrogen (N_2) molecules in the unit cell (as shown in Fig. S3(a)). Above 69 GPa, N_8H adopts a long zigzag chain structure with symmetric hydrogen bonds between the two nitrogen backbones, (as shown in Fig. S3(b)). Our calculations indicate that N_9H also adopts long zigzag chain structure at 40∼80 GPa, where only 1/3 of the zigzag chains have hydrogen attached on the nitrogen backbone chain (as shown in Fig. S3(c)).

Estimated from the internal energy difference between the molecular $R3m$ and ionic P-$43m$ type NH_5 structures (as shown in Fig. S3 (a)) constrained at 5 GPa (See Fig. S4 (a) and (b)), there is an ∼0.7 eV energy cost of a proton transfer from H_2 to NH_3 molecule, which is lower than energy cost of ∼0.9 eV[1] of forming NH_2^- and NH_4^+ ions in NH_3. With the same method, our calculation revealed an ∼1.0 eV energy cost of a proton transfer from N_2H_4 to NH_3 molecule, which is estimated from the internal energy difference between the ionic $C2$ and molecular $P1$ type N_3H_7 structures constrained at 5 GPa.

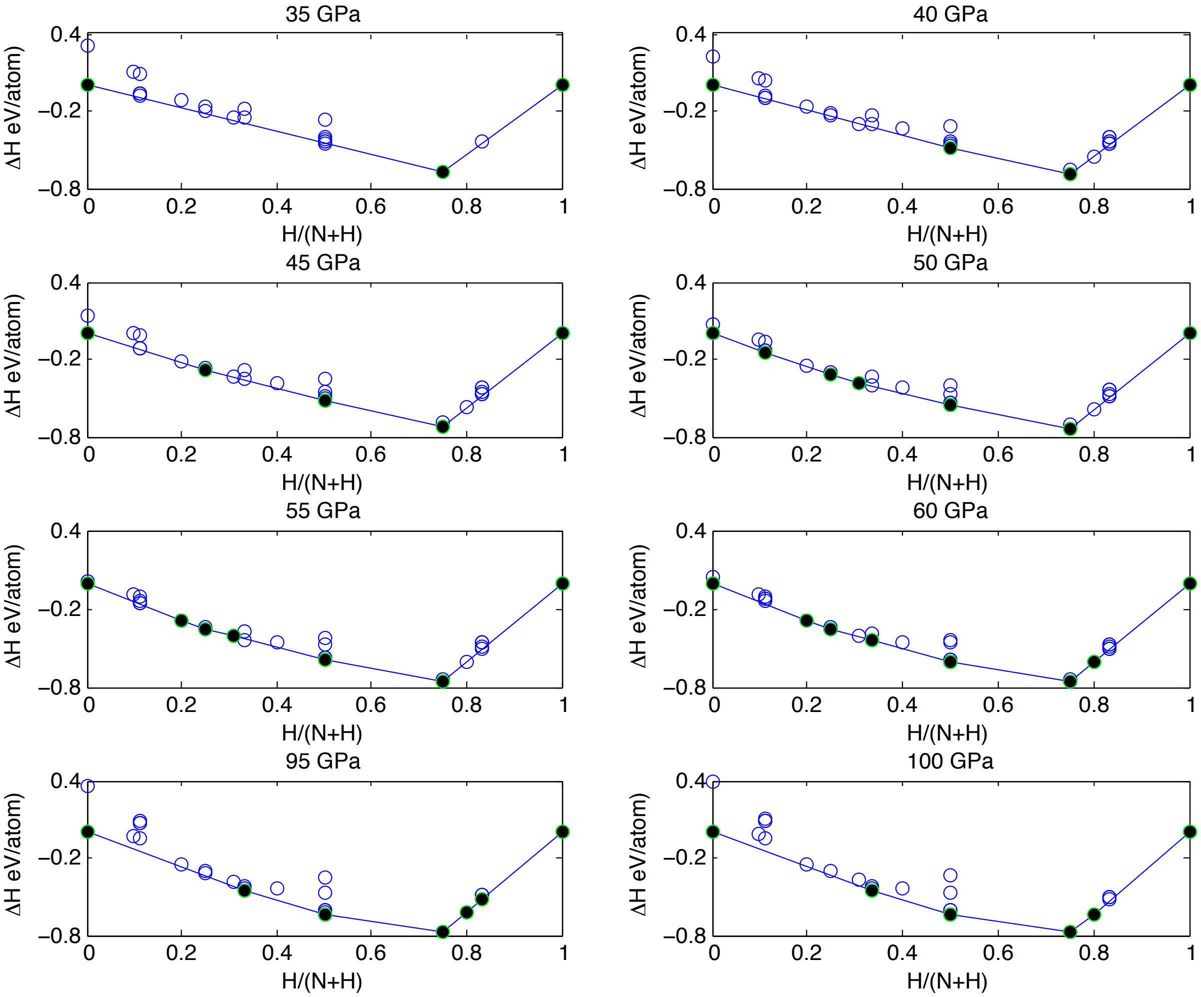

FIG. S1. (Color online) Convex hull for nitrogen hydride system at 35, 40, 45, 50, 55, 60, 95, 100 GPa. The solid and hollow symbols indicate stable and metastable phases, respectively

[1] Liebman, J. F. Existence and estimated enthalpies of formation of ammonium hydroxide, hydronium amide, and some related species. *Struct. Chem.* **8**, 313–315 (1997).

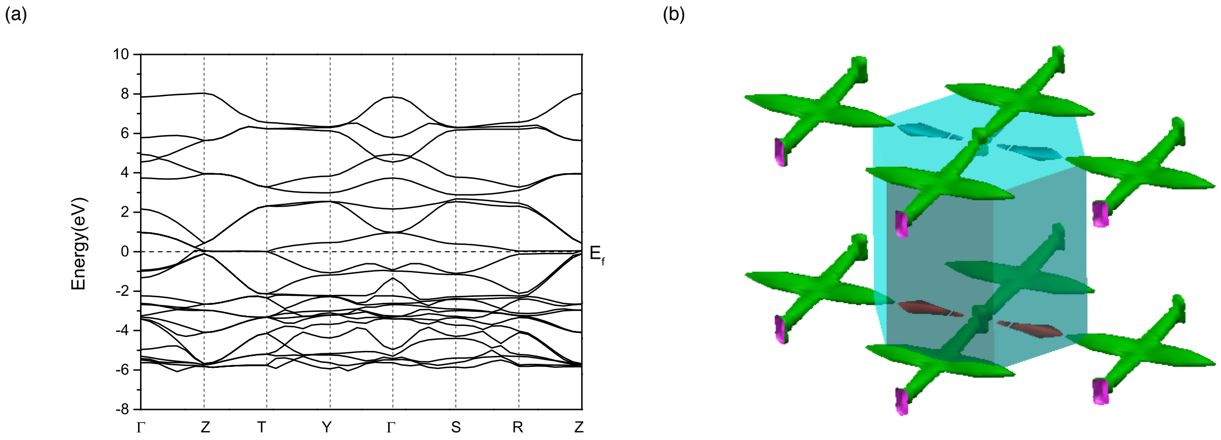

FIG. S2. (Color online) (a) Band structures of N_9H_4 and (b) Fermi surface of N_9H_4 at 60 GPa.

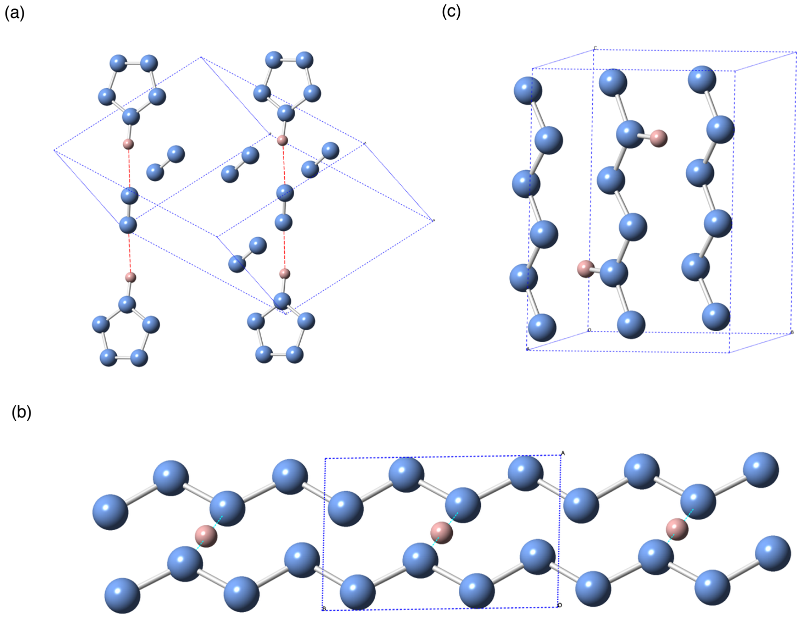

FIG. S3. (Color online) Structures for N_8H and N_9H, (a) $P\bar{1}$-N_8H molecular structure with four pentazole (N_5H) and six nitrogen molecules. (b) $P\bar{1}$-N_8H structure with zigzags-shaped nitrogen chain structure. (c) $Cc2m$-N_9H type with zigzags-shaped nitrogen chains. The cyan dot lines indicate symmetric hydrogen bonds.

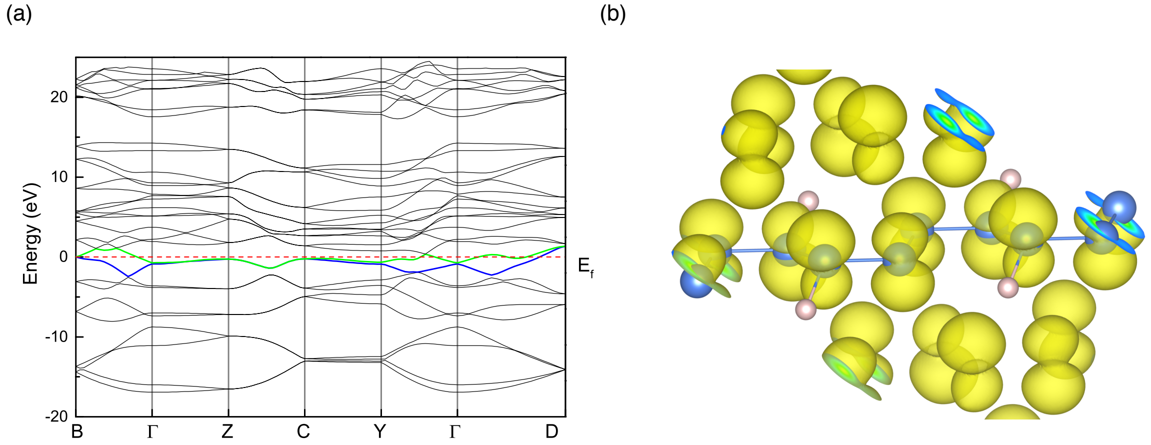

FIG. S4. (Color online) (a) The band structure of $P2_1/c$ N_2H structure at 120 GPa. (b) Electron density corresponding to the vicinity of the Fermi level for the $P2_1/c$ N_2H chain structure. The nitrogen atoms have sp^2 hybridization, and are responsible for a π^* orbits along the arm-chair nitrogen chain, which makes N_2H act as a metallic polymer.

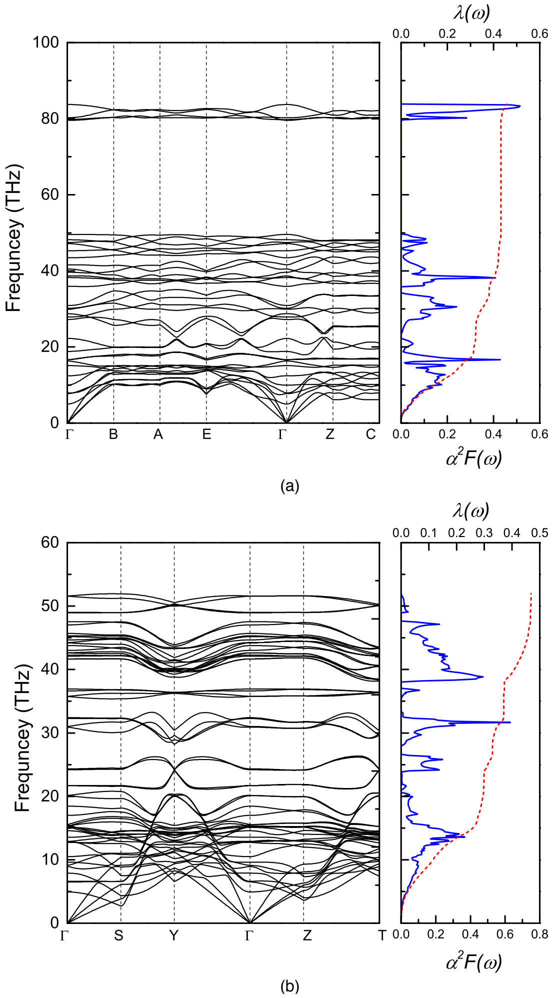

FIG. S5. (Color online) Phonon dispersion curves and Eliashberg phonon spectral function $\alpha F^2(\omega)$ together with the electron-phonon integral for (a) the $P2_1/c$ structure of N_2H at 60 GPa and (b) the $Cmc2_1$ structure of N_4H at 55 GPa

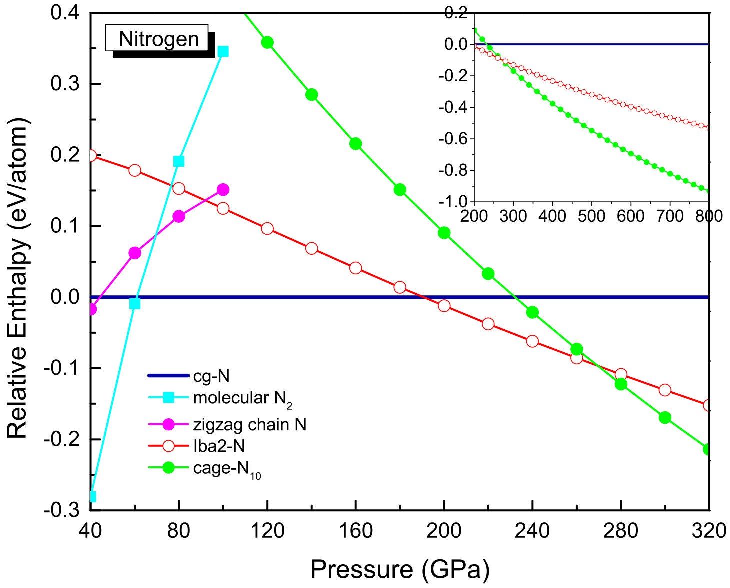

FIG. S6. (Color online) Enthalpy relative to cg-N as a function of pressure for nitrogen phases in the pressure range 40-800 GPa. The pink circle indicates that the zigzag chain structure found by the aniseed technique has competitive enthalpies to the molecular states and the singly bonded cg-N structure at 40-70 GPa.

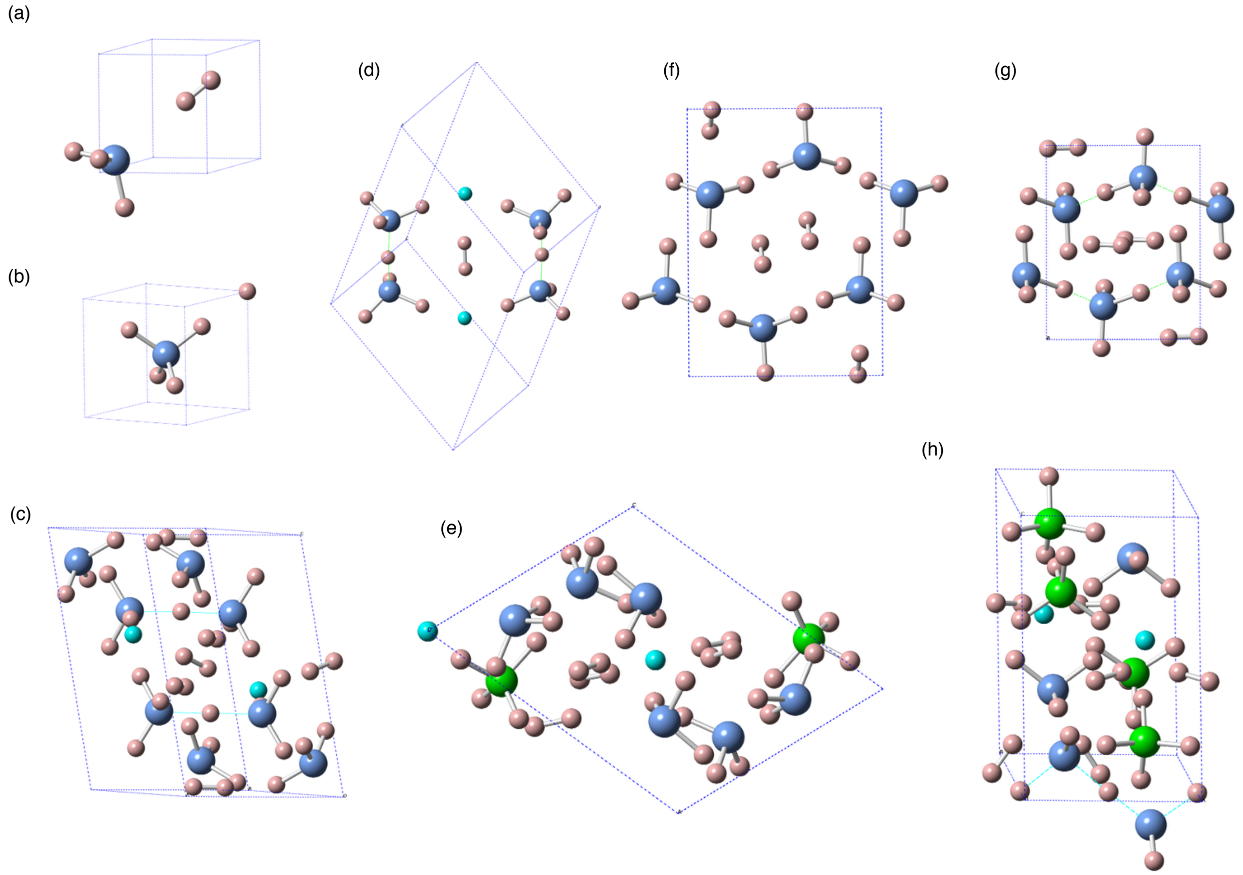

FIG. S7. (Color online) Various structures for NH_5. (a) Molecular $R3m$ structure at 5 GPa. (b) Ionic P-43m structure at 5 GPa. (c) Ionic $C2/c$ structure at 150 GPa. (d) The view of $[H_3N\cdots H\cdots NH_3]^+$ subunit in ionic $C2/c$ structure at 100 GPa. (e) Ionic $P2$ structure at 200 GPa. (f) Molecular $P2_1/c$ structure at 40 GPa, where hydrogen molecules locates at the empty channels formed by NH_3. (g) Another molecular $P2_1/c$ structure at 500 GPa. (h) Ionic $Ama2$ structure at 300 GPa. The small pink spheres indicate hydrogen atoms and the blue large spheres are nitrogen atoms. The nitrogen atom in the NH_4 cation and the H^- anion are noted with green and aqua spheres, respectively.

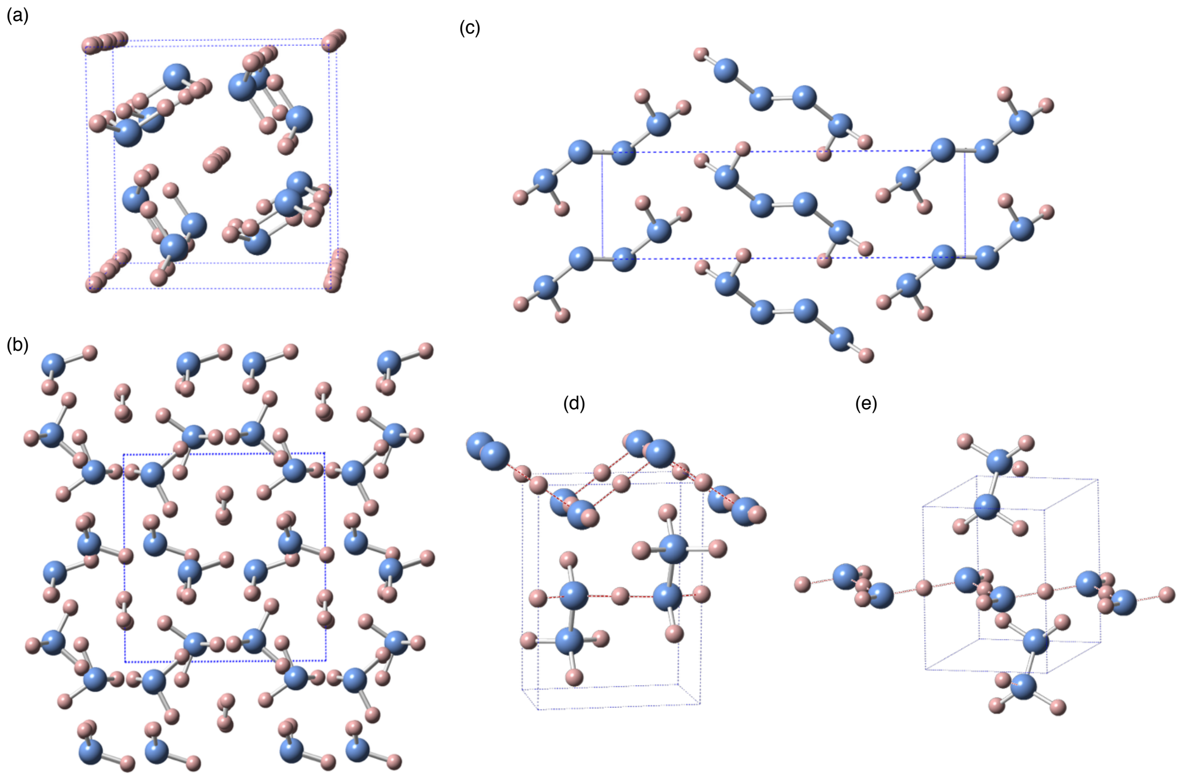

FIG. S8. (Color online) (a) Host-guest $I4/m$ NH_4 structure at 60 GPa, the hydrogen molecules fill channels in the frameworks formed by NH_3 molecules. (b) Host-guest $P2_1$ type NH_4 structure at 60 GPa. There is only small difference in the orientation directions of the NH_3 molecules between $P2_1$ and Pc type NH_4. (c) Short-chain molecular $P2_1/c$-NH structure at 36 GPa. (d) The $P2_1/m$-N_3H_7 structure (shown at 400 GPa) is stable in the range 380-680 GPa and consists of buckled $N_2H_5^+$ and NH_2^- layers. (e) Another $P2_1/m$ N_3H_7 structure (shown at 500 GPa, we call it $P2_1/m$-II), calculated to be stable above 680 GPa, is also an ionic phase consisting of NH_5^+ and NH_2^- ions. In contrasting to the $P2_1/m$ structure, the NH_2^- layers arrange as zigzag chains plain between the NH_5^+ layers. All the symmetric hydrogen bonds are shown by the red dashed lines.

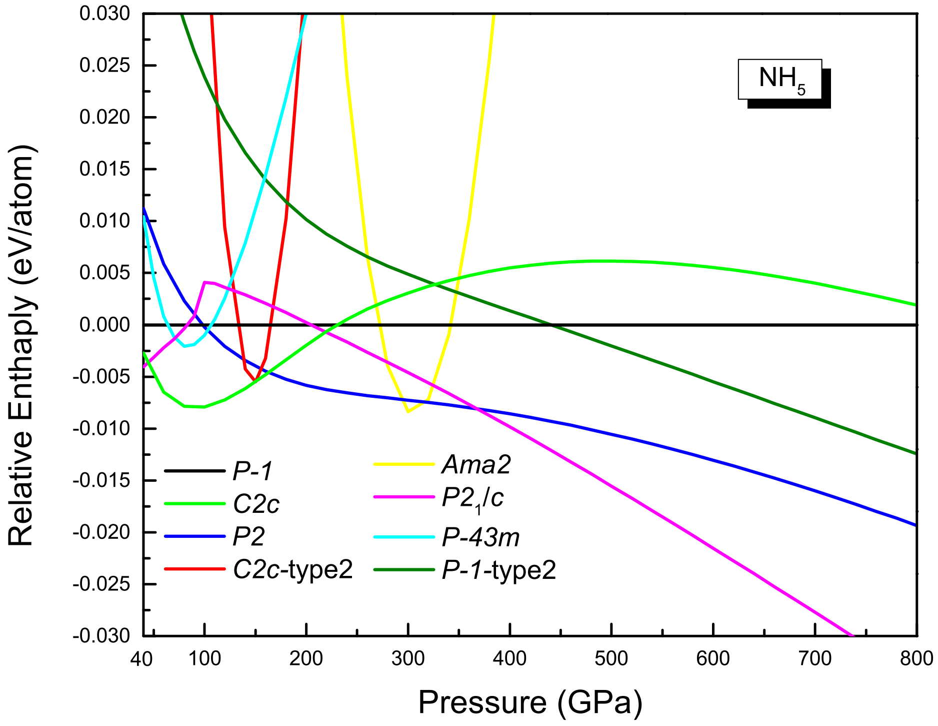

FIG. S9. (Color online) Enthalpy relative to P-1 as a function of pressure for NH_5 phases from 35-800 GPa. In the top figure, the clathrate $I4/m$, Pc, $C2/c$ and $P2_1$ structures have very close enthalpy to ionic $P1$ phase.

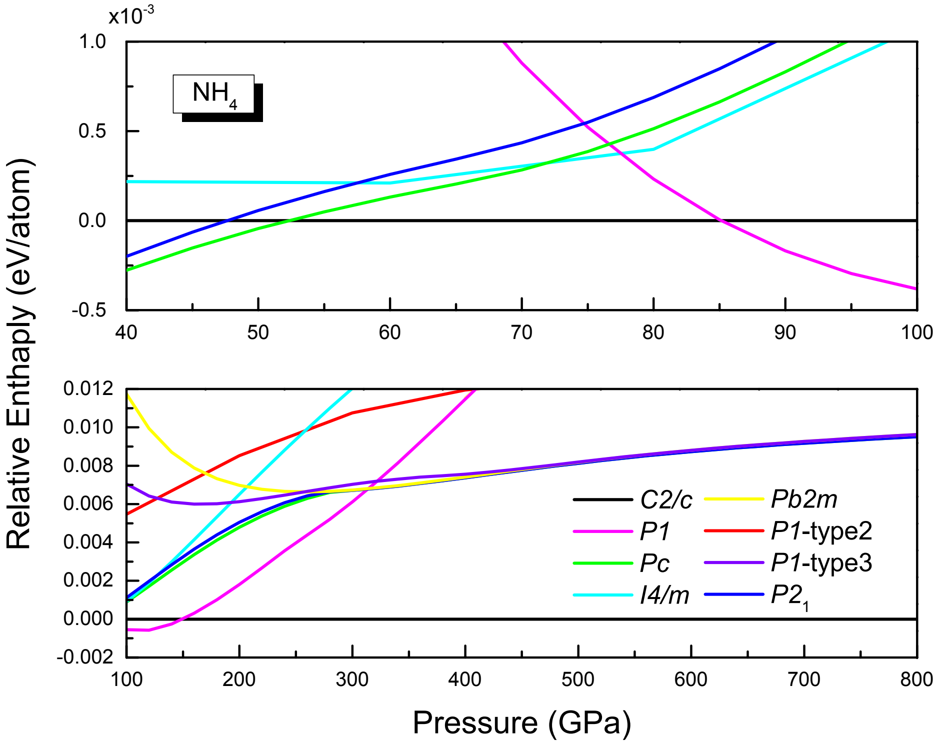

FIG. S10. (Color online) Enthalpy relative to $C2/c$ as a function of pressure for NH_4 phases from 40-800 GPa. In the top figure, the clathrate $I4/m$, Pc, $C2/c$ and $P2_1$ structures have very close enthalpy to ionic $P1$ phase. The NH_4 will adopt the same molecular type $C2/c$ structure above ∼150 GPa.

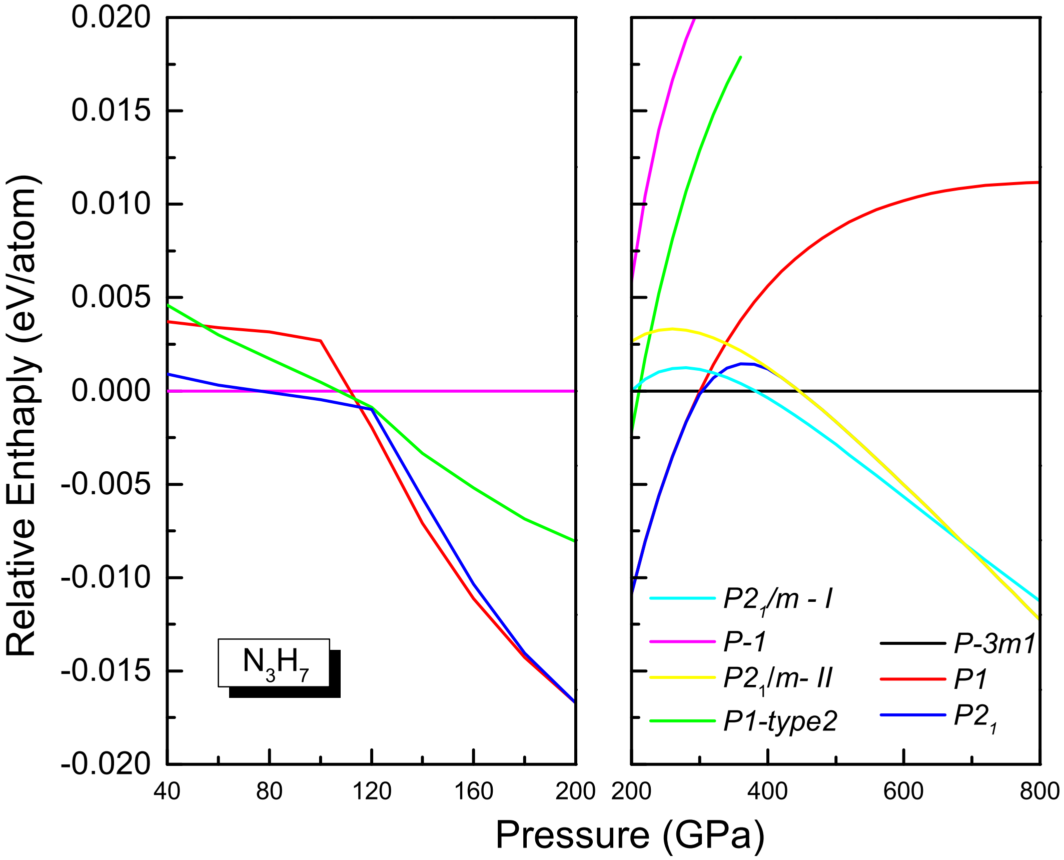

FIG. S11. (Color online) Enthalpy relative to $C2/c$ (left) and P-3m1 (right) as a function of pressure for N_3H_7 phases from 40-800 GPa.

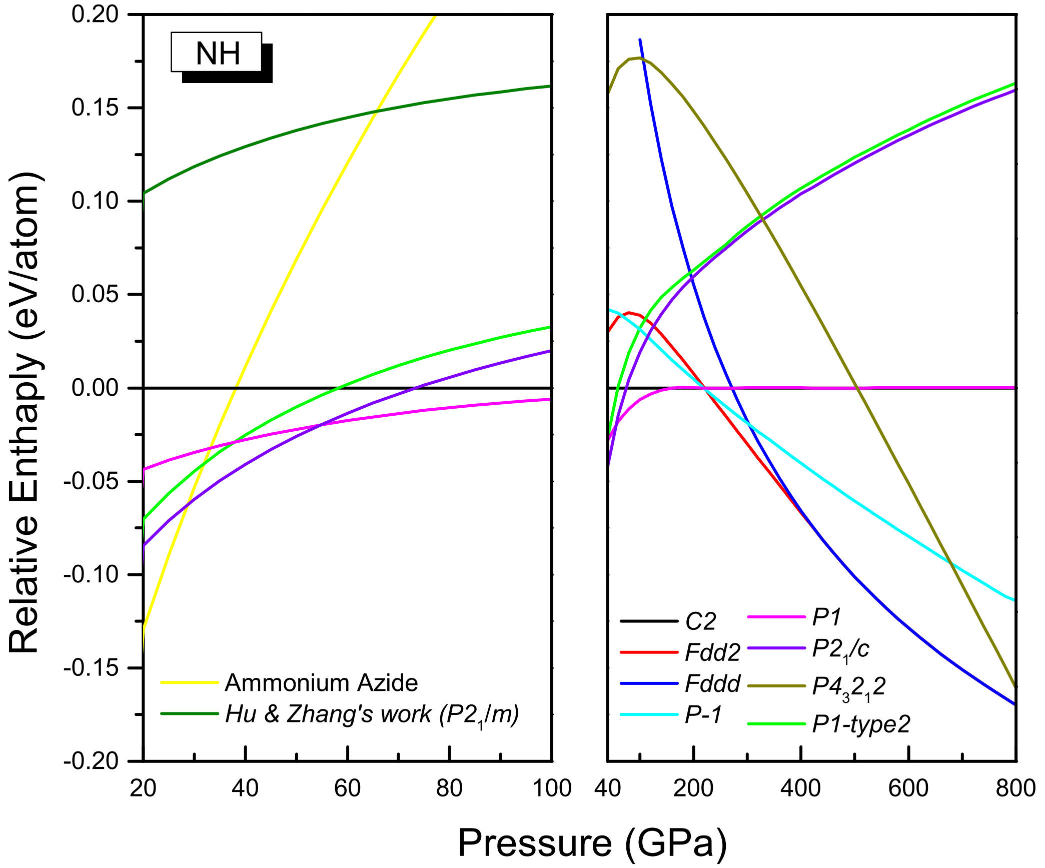

FIG. S12. (Color online) Enthalpy relative to $C2$ as a function of pressure for N_3H_7 phases from 20-800 GPa.

CIF files for all the phases discussed in this work:

```
data_N2H-P21c-60GPa
_audit_creation_date              2014-11-17
_audit_creation_method            'Materials Studio'
_symmetry_space_group_name_H-M    'P21/C'
_symmetry_Int_Tables_number       14
_symmetry_cell_setting            monoclinic
loop_
_symmetry_equiv_pos_as_xyz
  x,y,z
  -x,y+1/2,-z+1/2
  -x,-y,-z
  x,-y+1/2,z+1/2
_cell_length_a                    2.6437
_cell_length_b                    6.6085
_cell_length_c                    3.5311
_cell_angle_alpha                 90.0000
_cell_angle_beta                  109.3066
_cell_angle_gamma                 90.0000
loop_
_atom_site_label
_atom_site_type_symbol
_atom_site_fract_x
_atom_site_fract_y
_atom_site_fract_z
_atom_site_U_iso_or_equiv
_atom_site_adp_type
_atom_site_occupancy
H1     H    0.65535   0.21156   0.99276   0.00000  Uiso   1.00
N1     N    0.38452   0.40980   1.43830   0.00000  Uiso   1.00
N2     N    0.94757   0.91093   1.43417   0.00000  Uiso   1.00
```

```
data_N3H-P21c-60GPa
_audit_creation_date            2014-11-17
_audit_creation_method          'Materials Studio'
_symmetry_space_group_name_H-M   'P21/C'
_symmetry_Int_Tables_number      14
_symmetry_cell_setting           monoclinic
loop_
_symmetry_equiv_pos_as_xyz
  x,y,z
  -x,y+1/2,-z+1/2
  -x,-y,-z
  x,-y+1/2,z+1/2
_cell_length_a                 9.2859
_cell_length_b                 5.5501
_cell_length_c                 5.0172
_cell_angle_alpha              90.0000
_cell_angle_beta               138.3023
_cell_angle_gamma              90.0000
loop_
_atom_site_label
_atom_site_type_symbol
_atom_site_fract_x
_atom_site_fract_y
_atom_site_fract_z
_atom_site_U_iso_or_equiv
_atom_site_adp_type
_atom_site_occupancy
H1    H   -0.00852   0.46142   1.22601   0.00000  Uiso   1.00
H2    H   -0.49065   0.37859   0.77455   0.00000  Uiso   1.00
N1    N   -0.63109   0.45719   0.49203   0.00000  Uiso   1.00
N3    N   -0.12459   0.04024   0.98472   0.00000  Uiso   1.00
N9    N   -0.15761   0.26805   0.93137   0.00000  Uiso   1.00
N10    N   -0.72379   0.40187  -0.18278   0.00000  Uiso   1.00
N11    N   -0.78408   0.31946   0.19677   0.00000  Uiso   1.00
N12    N   -0.34718   0.18451   1.07396   0.00000  Uiso   1.00
```

data_N3H7-140GPa-P1

```
_audit_creation_date            2014-11-17
_audit_creation_method          'Materials Studio'
_symmetry_space_group_name_H-M    'P1'
_symmetry_Int_Tables_number      1
_symmetry_cell_setting          triclinic
loop_
_symmetry_equiv_pos_as_xyz
  x,y,z
_cell_length_a                  2.5262
_cell_length_b                  2.5278
_cell_length_c                  4.4879
_cell_angle_alpha               96.5798
_cell_angle_beta                83.4658
_cell_angle_gamma               81.7947
loop_
_atom_site_label
_atom_site_type_symbol
_atom_site_fract_x
_atom_site_fract_y
_atom_site_fract_z
_atom_site_U_iso_or_equiv
_atom_site_adp_type
_atom_site_occupancy
H1    H    0.51595  0.17110  0.02317  0.00000  Uiso   1.00
H2    H    0.21917  0.88079  0.27184  0.00000  Uiso   1.00
H3    H    0.62079  0.05587  0.50132  0.00000  Uiso   1.00
H4    H    0.93653  0.60981  0.02176  0.00000  Uiso   1.00
H5    H    0.06985  0.49478  0.50874  0.00000  Uiso   1.00
H6    H    0.65187  0.71526  0.76059  0.00000  Uiso   1.00
H7    H    0.16521  0.19982  0.77422  0.00000  Uiso   1.00
N1    N    0.64825  0.45595  0.15723  0.00000  Uiso   1.00
N2    N    0.89591  0.21025  0.36761  0.00000  Uiso   1.00
N3    N    0.25381  0.79695  0.75373  0.00000  Uiso   1.00
```

```
data_N3H7-200GPa-C2
_audit_creation_date              2014-11-17
_audit_creation_method            'Materials Studio'
_symmetry_space_group_name_H-M    'C2'
_symmetry_Int_Tables_number       5
_symmetry_cell_setting            monoclinic
loop_
_symmetry_equiv_pos_as_xyz
  x,y,z
  -x,y,-z
  x+1/2,y+1/2,z
  -x+1/2,y+1/2,-z
_cell_length_a                    3.1845
_cell_length_b                    3.6957
_cell_length_c                    5.8370
_cell_angle_alpha                 90.0000
_cell_angle_beta                  132.7539
_cell_angle_gamma                 90.0000
loop_
_atom_site_label
_atom_site_type_symbol
_atom_site_fract_x
_atom_site_fract_y
_atom_site_fract_z
_atom_site_U_iso_or_equiv
_atom_site_adp_type
_atom_site_occupancy
H1     H    -0.68731  -0.22025  -0.24349   0.00000  Uiso   1.00
H2     H    -0.67874   0.22892  -0.24324   0.00000  Uiso   1.00
H5     H    -1.24154   0.13919  -0.49313   0.00000  Uiso   1.00
N1     N    -0.48105  -0.00313  -0.10649   0.00000  Uiso   1.00
H6     H    -0.50000   0.49496  -0.00000   0.00000  Uiso   1.00
N2     N    -1.00000   0.47701  -0.50000   0.00000  Uiso   1.00
```

```
data_N3H7-P-3m1-380GPa
_audit_creation_date              2014-11-17
_audit_creation_method            'Materials Studio'
_symmetry_space_group_name_H-M    'P-3M1'
_symmetry_Int_Tables_number       164
_symmetry_cell_setting            trigonal
loop_
_symmetry_equiv_pos_as_xyz
  x,y,z
  -y,x-y,z
  -x+y,-x,z
  y,x,-z
  x-y,-y,-z
  -x,-x+y,-z
  -x,-y,-z
  y,-x+y,-z
  x-y,x,-z
  -y,-x,z
  -x+y,y,z
  x,x-y,z
_cell_length_a                    3.6109
_cell_length_b                    3.6109
_cell_length_c                    5.5005
_cell_angle_alpha                 90.0000
_cell_angle_beta                  90.0000
_cell_angle_gamma                 120.0000
loop_
_atom_site_label
_atom_site_type_symbol
_atom_site_fract_x
_atom_site_fract_y
_atom_site_fract_z
_atom_site_U_iso_or_equiv
_atom_site_adp_type
_atom_site_occupancy
H1     H     0.50000   0.50000   0.50000   0.00000  Uiso   1.00
H4     H     0.61279   0.80640   0.09549   0.00000  Uiso   1.00
H5     H     0.68759   0.84380   0.33402   0.00000  Uiso   1.00
H16    H     0.18589   0.81411   0.86287   0.00000  Uiso   1.00
N1     N     0.00000   0.00000   0.61719   0.00000  Uiso   1.00
```

```
N3    N    0.00000  0.00000  0.00000  0.00000  Uiso   1.00
N4    N    0.66667  0.33333  0.42905  0.00000  Uiso   1.00
N5    N    0.66667  0.33333  0.20078  0.00000  Uiso   1.00
N6    N    0.66667  0.33333  0.81549  0.00000  Uiso   1.00
```

data_N3H7-P21mI-500GPa

```
_audit_creation_date              2014-11-17
_audit_creation_method            'Materials Studio'
_symmetry_space_group_name_H-M    'P21/M'
_symmetry_Int_Tables_number       11
_symmetry_cell_setting            monoclinic
loop_
_symmetry_equiv_pos_as_xyz
  x,y,z
  -x,y+1/2,-z
  -x,-y,-z
  x,-y+1/2,z
_cell_length_a                    2.2082
_cell_length_b                    3.5510
_cell_length_c                    4.8272
_cell_angle_alpha                 90.0000
_cell_angle_beta                  88.8679
_cell_angle_gamma                 90.0000
loop_
_atom_site_label
_atom_site_type_symbol
_atom_site_fract_x
_atom_site_fract_y
_atom_site_fract_z
_atom_site_U_iso_or_equiv
_atom_site_adp_type
_atom_site_occupancy
H1    H    0.80584  0.47941  0.75381  0.00000  Uiso   1.00
H5    H    0.44658  0.25000  0.11546  0.00000  Uiso   1.00
N1    N    0.88268  0.25000  0.12729  0.00000  Uiso   1.00
N2    N    0.29650  0.25000  0.51255  0.00000  Uiso   1.00
N3    N    0.54736  0.25000  0.74233  0.00000  Uiso   1.00
H7    H    0.37397  0.75000  0.62742  0.00000  Uiso   1.00
H8    H    0.75979  0.75000  0.11672  0.00000  Uiso   1.00
H11    H    0.00000  0.50000  0.00000  0.00000  Uiso   1.00
H13    H    0.00000  0.00000  0.50000  0.00000  Uiso   1.00
```

```
data_N3H7-P21mII-700GPa
_audit_creation_date            2014-11-17
_audit_creation_method          'Materials Studio'
_symmetry_space_group_name_H-M    'P21/M'
_symmetry_Int_Tables_number      11
_symmetry_cell_setting           monoclinic
loop_
_symmetry_equiv_pos_as_xyz
  x,y,z
  -x,y+1/2,-z
  -x,-y,-z
  x,-y+1/2,z
_cell_length_a                 2.7632
_cell_length_b                 3.2247
_cell_length_c                 3.8442
_cell_angle_alpha              90.0000
_cell_angle_beta               80.0803
_cell_angle_gamma              90.0000
loop_
_atom_site_label
_atom_site_type_symbol
_atom_site_fract_x
_atom_site_fract_y
_atom_site_fract_z
_atom_site_U_iso_or_equiv
_atom_site_adp_type
_atom_site_occupancy
H1    H    0.80861   0.01344   0.24714   0.00000  Uiso   1.00
H2    H    0.31501   0.50416   0.25421   0.00000  Uiso   1.00
H9    H    0.59232   0.75000   0.45908   0.00000  Uiso   1.00
H10    H    0.25459   0.75000   0.00219   0.00000  Uiso   1.00
N1    N    0.87667   0.75000   0.11591   0.00000  Uiso   1.00
H13    H    0.00000   0.50000   0.50000   0.00000  Uiso   1.00
N3    N    0.36683   0.25000   0.11977   0.00000  Uiso   1.00
N4    N    0.75234   0.25000   0.49290   0.00000  Uiso   1.00
```

data_N4H-Cmc21-60GPa

```
_audit_creation_date            2014-11-17
_audit_creation_method          'Materials Studio'
_symmetry_space_group_name_H-M   'CMC21'
_symmetry_Int_Tables_number      36
_symmetry_cell_setting          orthorhombic
loop_
_symmetry_equiv_pos_as_xyz
  x,y,z
  -x,-y,z+1/2
  x,-y,z+1/2
  -x,y,z
  x+1/2,y+1/2,z
  -x+1/2,-y+1/2,z+1/2
  x+1/2,-y+1/2,z+1/2
  -x+1/2,y+1/2,z
_cell_length_a                4.2216
_cell_length_b                10.0796
_cell_length_c                5.1851
_cell_angle_alpha             90.0000
_cell_angle_beta              90.0000
_cell_angle_gamma             90.0000
loop_
_atom_site_label
_atom_site_type_symbol
_atom_site_fract_x
_atom_site_fract_y
_atom_site_fract_z
_atom_site_U_iso_or_equiv
_atom_site_adp_type
_atom_site_occupancy
N9    N   -0.24846  -0.20428  -0.84112   0.00000  Uiso   1.00
N11   N   -0.25124  -0.04572  -0.14553   0.00000  Uiso   1.00
H1    H   -0.50000  -0.12867  -0.50891   0.00000  Uiso   1.00
H3    H   -0.50000  -0.37856  -0.97819   0.00000  Uiso   1.00
N1    N   -0.50000  -0.07477  -0.27637   0.00000  Uiso   1.00
N2    N   -0.50000  -0.42599  -0.77360   0.00000  Uiso   1.00
N5    N   -0.50000  -0.17621  -0.71322   0.00000  Uiso   1.00
N3    N    0.00000  -0.17503  -0.71015   0.00000  Uiso   1.00
```

```
data_N8H-P-1-50GPa
_audit_creation_date              2014-11-17
_audit_creation_method            'Materials Studio'
_symmetry_space_group_name_H-M    'P-1'
_symmetry_Int_Tables_number       2
_symmetry_cell_setting            triclinic
loop_
_symmetry_equiv_pos_as_xyz
  x,y,z
  -x,-y,-z
_cell_length_a                    4.5270
_cell_length_b                    5.1046
_cell_length_c                    5.7911
_cell_angle_alpha                 109.1391
_cell_angle_beta                  101.3097
_cell_angle_gamma                 91.3480
loop_
_atom_site_label
_atom_site_type_symbol
_atom_site_fract_x
_atom_site_fract_y
_atom_site_fract_z
_atom_site_U_iso_or_equiv
_atom_site_adp_type
_atom_site_occupancy
H1     H    -0.91252   0.12423  -0.18585   0.00000  Uiso   1.00
N1     N    -1.46199   0.73857  -0.51497   0.00000  Uiso   1.00
N2     N    -1.53500   0.81098  -0.29307   0.00000  Uiso   1.00
N3     N    -1.21026   0.87114  -0.49457   0.00000  Uiso   1.00
N4     N    -1.31816   0.98241  -0.13295   0.00000  Uiso   1.00
N5     N    -1.12237   0.01678  -0.26028   0.00000  Uiso   1.00
N21    N    -0.57388   0.40174  -0.05155   0.00000  Uiso   1.00
N23    N    -1.07970   0.46563   0.65661   0.00000  Uiso   1.00
N24    N    -1.00768   0.60638   0.85326   0.00000  Uiso   1.00
loop_
_geom_bond_atom_site_label_1
_geom_bond_atom_site_label_2
_geom_bond_distance
_geom_bond_site_symmetry_2
_ccdc_geom_bond_type
```

H1	N5	1.024	.	S
N1	N2	1.326	.	S
N1	N3	1.276	.	D
N2	N4	1.288	.	D
N3	N5	1.290	1_565	S
N4	N5	1.301	1_565	S
N5	N3	1.290	1_545	S
N5	N4	1.301	1_545	S
N21	N21	1.109	2_465	T
N23	N24	1.107	.	T

```
data_N9H4-Ccc2-60GPa
_audit_creation_date            2014-11-17
_audit_creation_method          'Materials Studio'
_symmetry_space_group_name_H-M    'CCC2'
_symmetry_Int_Tables_number      37
_symmetry_cell_setting           orthorhombic
loop_
_symmetry_equiv_pos_as_xyz
  x,y,z
  -x,-y,z
  x,-y,z+1/2
  -x,y,z+1/2
  x+1/2,y+1/2,z
  -x+1/2,-y+1/2,z
  x+1/2,-y+1/2,z+1/2
  -x+1/2,y+1/2,z+1/2
_cell_length_a                9.7413
_cell_length_b                5.6071
_cell_length_c                4.8750
_cell_angle_alpha             90.0000
_cell_angle_beta              90.0000
_cell_angle_gamma             90.0000
loop_
_atom_site_label
_atom_site_type_symbol
_atom_site_fract_x
_atom_site_fract_y
_atom_site_fract_z
_atom_site_U_iso_or_equiv
_atom_site_adp_type
_atom_site_occupancy
H1    H   -0.41275  -0.52122  -0.27727   0.00000  Uiso   1.00
H3    H   -0.48871  -0.34740  -0.05480   0.00000  Uiso   1.00
N1    N   -0.27292  -0.35885  -0.92211   0.00000  Uiso   1.00
N2    N   -0.31571   0.23152  -0.92222   0.00000  Uiso   1.00
N3    N   -0.04283  -0.41108  -0.90691   0.00000  Uiso   1.00
N15    N   -0.33330   0.00045  -0.91749   0.00000  Uiso   1.00
N13    N   -0.50000  -0.50000  -0.16578   0.00000  Uiso   1.00
```

data_NH-C2-180GPa

```
_audit_creation_date            2014-11-17
_audit_creation_method          'Materials Studio'
_symmetry_space_group_name_H-M    'C2'
_symmetry_Int_Tables_number      5
_symmetry_cell_setting          monoclinic
loop_
_symmetry_equiv_pos_as_xyz
  x,y,z
  -x,y,-z
  x+1/2,y+1/2,z
  -x+1/2,y+1/2,-z
_cell_length_a                4.5168
_cell_length_b                10.2207
_cell_length_c                3.1632
_cell_angle_alpha              90.0000
_cell_angle_beta              148.2685
_cell_angle_gamma              90.0000
loop_
_atom_site_label
_atom_site_type_symbol
_atom_site_fract_x
_atom_site_fract_y
_atom_site_fract_z
_atom_site_U_iso_or_equiv
_atom_site_adp_type
_atom_site_occupancy
H1    H    0.07007   0.78398   0.35364   0.00000  Uiso   1.00
H2    H    0.05470   0.94859   0.32387   0.00000  Uiso   1.00
N1    N    0.17704   0.56191   0.87101   0.00000  Uiso   1.00
N2    N    0.34572   0.67938   0.15162   0.00000  Uiso   1.00
N3    N    0.31093   0.86781   0.61778   0.00000  Uiso   1.00
H9    H    0.00000   0.53972   0.00000   0.00000  Uiso   1.00
H11    H    0.50000   0.86724   0.50000   0.00000  Uiso   1.00
```

```
data_NH-Fdd2-400GPa
_audit_creation_date            2014-11-17
_audit_creation_method          'Materials Studio'
_symmetry_space_group_name_H-M    'FDD2'
_symmetry_Int_Tables_number      43
_symmetry_cell_setting          orthorhombic
loop_
_symmetry_equiv_pos_as_xyz
  x,y,z
  -x,-y,z
  x+1/4,-y+1/4,z+1/4
  -x+1/4,y+1/4,z+1/4
  x,y+1/2,z+1/2
  -x,-y+1/2,z+1/2
  x+1/4,-y+3/4,z+3/4
  -x+1/4,y+3/4,z+3/4
  x+1/2,y,z+1/2
  -x+1/2,-y,z+1/2
  x+3/4,-y+1/4,z+3/4
  -x+3/4,y+1/4,z+3/4
  x+1/2,y+1/2,z
  -x+1/2,-y+1/2,z
  x+3/4,-y+3/4,z+1/4
  -x+3/4,y+3/4,z+1/4
_cell_length_a                2.3327
_cell_length_b                11.7381
_cell_length_c                5.8368
_cell_angle_alpha              90.0000
_cell_angle_beta               90.0000
_cell_angle_gamma              90.0000
loop_
_atom_site_label
_atom_site_type_symbol
_atom_site_fract_x
_atom_site_fract_y
_atom_site_fract_z
_atom_site_U_iso_or_equiv
_atom_site_adp_type
_atom_site_occupancy
H1    H    0.12088   0.87726   0.46953   0.00000  Uiso   1.00
```

```
H2    H    0.72229   0.80429   0.35999   0.00000  Uiso   1.00
N1    N    0.64813   0.95062   0.24733   0.00000  Uiso   1.00
N2    N    0.65209   0.54874   0.44790   0.00000  Uiso   1.00
```

data_NH-Fddd-460GPa

```
_audit_creation_date              2014-11-17
_audit_creation_method            'Materials Studio'
_symmetry_space_group_name_H-M    'FDDD'
_symmetry_Int_Tables_number       70
_symmetry_cell_setting            orthorhombic
loop_
_symmetry_equiv_pos_as_xyz
  x,y,z
  -x,-y,z
  -x,y,-z
  x,-y,-z
  -x+1/4,-y+1/4,-z+1/4
  x+1/4,y+1/4,-z+1/4
  x+1/4,-y+1/4,z+1/4
  -x+1/4,y+1/4,z+1/4
  x,y+1/2,z+1/2
  -x,-y+1/2,z+1/2
  -x,y+1/2,-z+1/2
  x,-y+1/2,-z+1/2
  -x+1/4,-y+3/4,-z+3/4
  x+1/4,y+3/4,-z+3/4
  x+1/4,-y+3/4,z+3/4
  -x+1/4,y+3/4,z+3/4
  x+1/2,y,z+1/2
  -x+1/2,-y,z+1/2
  -x+1/2,y,-z+1/2
  x+1/2,-y,-z+1/2
  -x+3/4,-y+1/4,-z+3/4
  x+3/4,y+1/4,-z+3/4
  x+3/4,-y+1/4,z+3/4
  -x+3/4,y+1/4,z+3/4
  x+1/2,y+1/2,z
  -x+1/2,-y+1/2,z
  -x+1/2,y+1/2,-z
  x+1/2,-y+1/2,-z
  -x+3/4,-y+3/4,-z+1/4
  x+3/4,y+3/4,-z+1/4
  x+3/4,-y+3/4,z+1/4
  -x+3/4,y+3/4,z+1/4
```

```
_cell_length_a                2.2939
_cell_length_b                5.7566
_cell_length_c                11.6066
_cell_angle_alpha             90.0000
_cell_angle_beta              90.0000
_cell_angle_gamma             90.0000
loop_
_atom_site_label
_atom_site_type_symbol
_atom_site_fract_x
_atom_site_fract_y
_atom_site_fract_z
_atom_site_U_iso_or_equiv
_atom_site_adp_type
_atom_site_occupancy
N1    N    1.09849   0.89887   0.20080   0.00000  Uiso   1.00
H1    H    0.62500   0.12500   0.12500   0.00000  Uiso   1.00
H2    H    1.00000  -0.00000   0.05487   0.00000  Uiso   1.00
```

```
data_NH-P1-55GPa
_audit_creation_date            2014-11-17
_audit_creation_method          'Materials Studio'
_symmetry_space_group_name_H-M    'P1'
_symmetry_Int_Tables_number      1
_symmetry_cell_setting          triclinic
loop_
_symmetry_equiv_pos_as_xyz
  x,y,z
_cell_length_a                 2.8058
_cell_length_b                 3.4848
_cell_length_c                 5.8044
_cell_angle_alpha              102.9249
_cell_angle_beta               96.7369
_cell_angle_gamma              109.0493
loop_
_atom_site_label
_atom_site_type_symbol
_atom_site_fract_x
_atom_site_fract_y
_atom_site_fract_z
_atom_site_U_iso_or_equiv
_atom_site_adp_type
_atom_site_occupancy
H1   H   0.30150  0.99598  0.64632  0.00000  Uiso  1.00
H2   H   0.23292  0.58330  0.96020  0.00000  Uiso  1.00
H3   H   0.44275  0.16116  0.95793  0.00000  Uiso  1.00
H4   H   0.61578  0.22209  0.45279  0.00000  Uiso  1.00
H5   H   0.75238  0.86531  0.80309  0.00000  Uiso  1.00
H6   H   0.13148  0.43898  0.65720  0.00000  Uiso  1.00
N1   N   0.80966  0.83537  0.19141  0.00000  Uiso  1.00
N2   N   0.80337  0.79153  0.40573  0.00000  Uiso  1.00
N3   N   0.96071  0.49239  0.80329  0.00000  Uiso  1.00
N4   N   0.59650  0.10991  0.80101  0.00000  Uiso  1.00
N5   N   0.43472  0.41617  0.40341  0.00000  Uiso  1.00
N6   N   0.13523  0.18559  0.18039  0.00000  Uiso  1.00
```

data_NH-P21c-50GPa

```
_audit_creation_date              2014-11-17
_audit_creation_method            'Materials Studio'
_symmetry_space_group_name_H-M    'P21/C'
_symmetry_Int_Tables_number       14
_symmetry_cell_setting            monoclinic
loop_
_symmetry_equiv_pos_as_xyz
  x,y,z
  -x,y+1/2,-z+1/2
  -x,-y,-z
  x,-y+1/2,z+1/2
_cell_length_a                    2.7818
_cell_length_b                    9.3022
_cell_length_c                    3.5365
_cell_angle_alpha                 90.0000
_cell_angle_beta                  128.3939
_cell_angle_gamma                 90.0000
loop_
_atom_site_label
_atom_site_type_symbol
_atom_site_fract_x
_atom_site_fract_y
_atom_site_fract_z
_atom_site_U_iso_or_equiv
_atom_site_adp_type
_atom_site_occupancy
H1     H     0.45360   0.72754  -0.47348   0.00000  Uiso   1.00
H2     H    -0.91580   0.61136  -0.95002   0.00000  Uiso   1.00
N1     N     0.08324   0.65792  -0.68296   0.00000  Uiso   1.00
N2     N     0.09555   0.56005  -0.40070   0.00000  Uiso   1.00
```

```
data_NH2-C2c-200GPa
_audit_creation_date              2014-11-17
_audit_creation_method            'Materials Studio'
_symmetry_space_group_name_H-M    'C2/C'
_symmetry_Int_Tables_number       15
_symmetry_cell_setting            monoclinic
loop_
_symmetry_equiv_pos_as_xyz
  x,y,z
  -x,y,-z+1/2
  -x,-y,-z
  x,-y,z+1/2
  x+1/2,y+1/2,z
  -x+1/2,y+1/2,-z+1/2
  -x+1/2,-y+1/2,-z
  x+1/2,-y+1/2,z+1/2
_cell_length_a                    8.5377
_cell_length_b                    3.2422
_cell_length_c                    8.4095
_cell_angle_alpha                 90.0000
_cell_angle_beta                  147.8054
_cell_angle_gamma                 90.0000
loop_
_atom_site_label
_atom_site_type_symbol
_atom_site_fract_x
_atom_site_fract_y
_atom_site_fract_z
_atom_site_U_iso_or_equiv
_atom_site_adp_type
_atom_site_occupancy
H1    H    0.30189  -0.01973   0.43863   0.00000  Uiso   1.00
H2    H    0.42563  -0.36395   0.37801   0.00000  Uiso   1.00
H3    H    0.62562  -0.12741   0.66148   0.00000  Uiso   1.00
N1    N    0.39326   0.33840   0.52918   0.00000  Uiso   1.00
N2    N    0.28796   0.15217   0.15524   0.00000  Uiso   1.00
H13   H    1.00000   0.35972   0.75000   0.00000  Uiso   1.00
H15   H    0.50000   0.64535   0.25000   0.00000  Uiso   1.00
```

```
data_NH4-C2c-60GPa
_audit_creation_date            2014-11-17
_audit_creation_method           'Materials Studio'
_symmetry_space_group_name_H-M    'C2/C'
_symmetry_Int_Tables_number      15
_symmetry_cell_setting           monoclinic
loop_
_symmetry_equiv_pos_as_xyz
  x,y,z
  -x,y,-z+1/2
  -x,-y,-z
  x,-y,z+1/2
  x+1/2,y+1/2,z
  -x+1/2,y+1/2,-z+1/2
  -x+1/2,-y+1/2,-z
  x+1/2,-y+1/2,z+1/2
_cell_length_a                7.6956
_cell_length_b                4.4793
_cell_length_c                7.6953
_cell_angle_alpha              90.0000
_cell_angle_beta               90.0237
_cell_angle_gamma              90.0000
loop_
_atom_site_label
_atom_site_type_symbol
_atom_site_fract_x
_atom_site_fract_y
_atom_site_fract_z
_atom_site_U_iso_or_equiv
_atom_site_adp_type
_atom_site_occupancy
H1    H    0.04019   0.75351   0.53774   0.00000  Uiso   1.00
H2    H    0.74679   0.93117   1.00106   0.00000  Uiso   1.00
H3    H    0.86725   0.07620   1.18303   0.00000  Uiso   1.00
H4    H    0.21230   0.75196   1.29012   0.00000  Uiso   1.00
H5    H    0.75520   0.09311   0.99599   0.00000  Uiso   1.00
H8    H    0.06732   0.57461   1.11625   0.00000  Uiso   1.00
H9    H    0.93952   0.07071   0.87620   0.00000  Uiso   1.00
H10    H    0.37249   0.06912   0.68887   0.00000  Uiso   1.00
N1    N    0.84221   0.25054   1.26218   0.00000  Uiso   1.00
```

N3 N 0.98779 0.25103 0.59218 0.00000 Uiso 1.00

data_NH4-I4m-50GPa
_audit_creation_date 2014-11-17
_audit_creation_method 'Materials Studio'
_symmetry_space_group_name_H-M 'I4/M'
_symmetry_Int_Tables_number 87
_symmetry_cell_setting tetragonal
loop_
_symmetry_equiv_pos_as_xyz
x,y,z
-x,-y,z
-y,x,z
y,-x,z
-x,-y,-z
x,y,-z
y,-x,-z
-y,x,-z
x+1/2,y+1/2,z+1/2
-x+1/2,-y+1/2,z+1/2
-y+1/2,x+1/2,z+1/2
y+1/2,-x+1/2,z+1/2
-x+1/2,-y+1/2,-z+1/2
x+1/2,y+1/2,-z+1/2
y+1/2,-x+1/2,-z+1/2
-y+1/2,x+1/2,-z+1/2
_cell_length_a 4.2902
_cell_length_b 4.2902
_cell_length_c 3.5802
_cell_angle_alpha 90.0000
_cell_angle_beta 90.0000
_cell_angle_gamma 90.0000
loop_
_atom_site_label
_atom_site_type_symbol
_atom_site_fract_x
_atom_site_fract_y
_atom_site_fract_z
_atom_site_U_iso_or_equiv
_atom_site_adp_type
_atom_site_occupancy
H2 H 0.02078 0.71190 -0.20640 0.00000 Uiso 1.00

```
H1    H   -0.13773   0.27429  -0.50000   0.00000  Uiso   1.00
H4    H    0.00000   0.00000  -0.65047   0.00000  Uiso   1.00
H8    H    0.00000   0.00000  -0.84333   0.00000  Uiso   1.00
N1    N    0.33850   0.16352  -0.00000   0.00000  Uiso   1.00
```

data_NH4-P1-100GPa

```
_audit_creation_date              2014-11-17
_audit_creation_method            'Materials Studio'
_symmetry_space_group_name_H-M    'P1'
_symmetry_Int_Tables_number       1
_symmetry_cell_setting            triclinic
loop_
_symmetry_equiv_pos_as_xyz
  x,y,z
_cell_length_a                    4.1935
_cell_length_b                    4.2892
_cell_length_c                    6.6560
_cell_angle_alpha                 99.4034
_cell_angle_beta                  79.7875
_cell_angle_gamma                 104.0809
loop_
_atom_site_label
_atom_site_type_symbol
_atom_site_fract_x
_atom_site_fract_y
_atom_site_fract_z
_atom_site_U_iso_or_equiv
_atom_site_adp_type
_atom_site_occupancy
H1    H    0.40878   0.38311   0.81846   0.00000  Uiso   1.00
H2    H    0.58662   0.78855   0.90315   0.00000  Uiso   1.00
H3    H    0.15900   0.16458   0.98296   0.00000  Uiso   1.00
H4    H    0.81505   0.55115   0.78567   0.00000  Uiso   1.00
H5    H    0.83038   0.32420   0.24226   0.00000  Uiso   1.00
H6    H    0.76118   0.05813   0.65081   0.00000  Uiso   1.00
H7    H    0.16391   0.96734   0.58283   0.00000  Uiso   1.00
H8    H    0.58959   0.35244   0.59654   0.00000  Uiso   1.00
H9    H    0.65740   0.54896   0.04696   0.00000  Uiso   1.00
H10   H    0.06706   0.26213   0.40315   0.00000  Uiso   1.00
H11   H    0.50110   0.43510   0.36462   0.00000  Uiso   1.00
H12   H    0.21770   0.13925   0.60770   0.00000  Uiso   1.00
H13   H    0.40508   0.01074   0.79033   0.00000  Uiso   1.00
H14   H    0.98735   0.91199   0.85094   0.00000  Uiso   1.00
H15   H    0.84258   0.29401   0.86406   0.00000  Uiso   1.00
H16   H    0.81938   0.71569   0.54441   0.00000  Uiso   1.00
```

H17	H	0.00725	0.29552	0.80837	0.00000	Uiso	1.00
H18	H	0.34971	0.72970	0.16146	0.00000	Uiso	1.00
H19	H	0.06516	0.86309	0.08494	0.00000	Uiso	1.00
H20	H	0.12415	0.65092	0.37207	0.00000	Uiso	1.00
H21	H	0.68029	0.93631	0.20848	0.00000	Uiso	1.00
H22	H	0.60683	0.02987	0.44452	0.00000	Uiso	1.00
H23	H	0.68826	0.59303	0.15236	0.00000	Uiso	1.00
H24	H	0.42119	0.74860	0.54946	0.00000	Uiso	1.00
H25	H	0.65117	0.14695	0.04180	0.00000	Uiso	1.00
H26	H	0.32155	0.28062	0.16206	0.00000	Uiso	1.00
H27	H	0.08510	0.48022	0.59823	0.00000	Uiso	1.00
H28	H	0.26361	0.67044	0.79747	0.00000	Uiso	1.00
H29	H	0.34904	0.03759	0.32573	0.00000	Uiso	1.00
H30	H	0.05856	0.49154	0.05198	0.00000	Uiso	1.00
H31	H	0.44152	0.79779	0.34091	0.00000	Uiso	1.00
H32	H	0.92008	0.88189	0.33447	0.00000	Uiso	1.00
N1	N	0.30534	0.26351	0.31227	0.00000	Uiso	1.00
N2	N	0.78837	0.77832	0.79336	0.00000	Uiso	1.00
N3	N	0.74461	0.19562	0.54348	0.00000	Uiso	1.00
N4	N	0.21724	0.70559	0.04221	0.00000	Uiso	1.00
N5	N	0.81822	0.12553	0.13213	0.00000	Uiso	1.00
N6	N	0.39572	0.20029	0.89849	0.00000	Uiso	1.00
N7	N	0.30850	0.58124	0.64449	0.00000	Uiso	1.00
N8	N	0.88277	0.67016	0.38754	0.00000	Uiso	1.00

data_NH4-P21-60GPa

```
_audit_creation_date            2014-11-17
_audit_creation_method          'Materials Studio'
_symmetry_space_group_name_H-M    'P21'
_symmetry_Int_Tables_number      4
_symmetry_cell_setting          monoclinic
loop_
_symmetry_equiv_pos_as_xyz
  x,y,z
  -x,y+1/2,-z
_cell_length_a                4.5052
_cell_length_b                5.3454
_cell_length_c                5.5148
_cell_angle_alpha             90.0000
_cell_angle_beta              88.3263
_cell_angle_gamma             90.0000
loop_
_atom_site_label
_atom_site_type_symbol
_atom_site_fract_x
_atom_site_fract_y
_atom_site_fract_z
_atom_site_U_iso_or_equiv
_atom_site_adp_type
_atom_site_occupancy
H1    H    0.31608  0.31817  0.68547  0.00000  Uiso   1.00
H2    H    0.68921  0.04090  0.08231  0.00000  Uiso   1.00
H3    H    0.79203  0.99938  0.69507  0.00000  Uiso   1.00
H4    H    0.30554  0.00748  0.80800  0.00000  Uiso   1.00
H5    H    0.18464  0.13125  0.32708  0.00000  Uiso   1.00
H6    H    0.66984  0.29626  0.67038  0.00000  Uiso   1.00
H7    H    0.68722  0.99473  0.79830  0.00000  Uiso   1.00
H8    H    0.82625  0.45817  0.91924  0.00000  Uiso   1.00
H9    H    0.20907  0.99268  0.70493  0.00000  Uiso   1.00
H10    H    0.17544  0.64009  0.67358  0.00000  Uiso   1.00
H11    H    0.00389  0.33560  0.49094  0.00000  Uiso   1.00
H12    H    0.97177  0.78111  0.91380  0.00000  Uiso   1.00
H13    H    0.50164  0.72992  0.73546  0.00000  Uiso   1.00
H14    H    0.49170  0.78425  0.02449  0.00000  Uiso   1.00
H15    H    0.98179  0.19837  0.81293  0.00000  Uiso   1.00
```

H16	H	0.49209	0.00759	0.49009	0.00000	Uiso	1.00
N1	N	0.98422	0.85004	0.08267	0.00000	Uiso	1.00
N2	N	0.99962	0.65388	0.56751	0.00000	Uiso	1.00
N3	N	0.50691	0.64351	0.90331	0.00000	Uiso	1.00
N4	N	0.50672	0.82575	0.43205	0.00000	Uiso	1.00

```
data_NH4-P21c-500GPa
_audit_creation_date              2014-11-17
_audit_creation_method            'Materials Studio'
_symmetry_space_group_name_H-M    'P21/C'
_symmetry_Int_Tables_number       14
_symmetry_cell_setting            monoclinic
loop_
_symmetry_equiv_pos_as_xyz
  x,y,z
  -x,y+1/2,-z+1/2
  -x,-y,-z
  x,-y+1/2,z+1/2
_cell_length_a                    2.5326
_cell_length_b                    3.5714
_cell_length_c                    4.5278
_cell_angle_alpha                 90.0000
_cell_angle_beta                  90.8158
_cell_angle_gamma                 90.0000
loop_
_atom_site_label
_atom_site_type_symbol
_atom_site_fract_x
_atom_site_fract_y
_atom_site_fract_z
_atom_site_U_iso_or_equiv
_atom_site_adp_type
_atom_site_occupancy
H1     H     0.72418   0.29531   0.98785   0.00000  Uiso   1.00
H2     H     0.20674   0.14193   0.96158   0.00000  Uiso   1.00
H3     H     0.75677   0.48713   0.98242   0.00000  Uiso   1.00
H4     H     0.09027   0.62311   1.23476   0.00000  Uiso   1.00
H5     H     0.47336   0.88792   1.24026   0.00000  Uiso   1.00
N1     N     0.74237   0.87098   0.82676   0.00000  Uiso   1.00
```

data_NH4-Pc-50GPa

```
_audit_creation_date            2014-11-17
_audit_creation_method          'Materials Studio'
_symmetry_space_group_name_H-M    'PC'
_symmetry_Int_Tables_number      7
_symmetry_cell_setting          monoclinic
loop_
_symmetry_equiv_pos_as_xyz
  x,y,z
  x,-y,z+1/2
_cell_length_a                 7.1286
_cell_length_b                 5.6118
_cell_length_c                 5.4375
_cell_angle_alpha              90.0000
_cell_angle_beta               139.9399
_cell_angle_gamma              90.0000
loop_
_atom_site_label
_atom_site_type_symbol
_atom_site_fract_x
_atom_site_fract_y
_atom_site_fract_z
_atom_site_U_iso_or_equiv
_atom_site_adp_type
_atom_site_occupancy
H1    H   -0.36122   0.67208  -0.08000   0.00000  Uiso   1.00
H2    H   -0.70294   0.08204  -0.67922   0.00000  Uiso   1.00
H3    H   -0.82483   0.69619   0.16619   0.00000  Uiso   1.00
H4    H   -0.33519   0.18605   0.15514   0.00000  Uiso   1.00
H5    H   -0.20551   0.31780  -0.08532   0.00000  Uiso   1.00
H6    H   -0.70898   0.67884  -0.40953   0.00000  Uiso   1.00
H7    H   -0.72654   0.79688   0.24758   0.00000  Uiso   1.00
H8    H   -0.24420   0.29244   0.22921   0.00000  Uiso   1.00
H17   H   -0.19006   0.08117   0.75397   0.00000  Uiso   1.00
H18   H   -0.03758   0.48634   0.28121   0.00000  Uiso   1.00
H19   H   -0.99878   0.91960  -0.23902   0.00000  Uiso   1.00
H21   H   -0.52604   0.73676   0.18523   0.00000  Uiso   1.00
H22   H   -0.52140   0.02212   0.24282   0.00000  Uiso   1.00
H25   H   -0.04857   0.80738   0.14153   0.00000  Uiso   1.00
H26   H   -0.85100   0.34037  -0.73329   0.00000  Uiso   1.00
```

H29	H	-0.52340	0.49004	0.46399	0.00000	Uiso	1.00
N1	N	-0.00689	0.08383	0.82752	0.00000	Uiso	1.00
N2	N	-0.03682	0.56762	0.60185	0.00000	Uiso	1.00
N5	N	-0.51264	0.90159	0.11402	0.00000	Uiso	1.00
N6	N	-0.53530	0.43307	0.27314	0.00000	Uiso	1.00

```
data_NH5-Ama2-300GPa
_audit_creation_date              2014-11-17
_audit_creation_method            'Materials Studio'
_symmetry_space_group_name_H-M    'AMA2'
_symmetry_Int_Tables_number       40
_symmetry_cell_setting            orthorhombic
loop_
_symmetry_equiv_pos_as_xyz
  x,y,z
  -x,-y,z
  x+1/2,-y,z
  -x+1/2,y,z
  x,y+1/2,z+1/2
  -x,-y+1/2,z+1/2
  x+1/2,-y+1/2,z+1/2
  -x+1/2,y+1/2,z+1/2
_cell_length_a                    3.6201
_cell_length_b                    10.8153
_cell_length_c                    4.6307
_cell_angle_alpha                 90.0000
_cell_angle_beta                  90.0000
_cell_angle_gamma                 90.0000
loop_
_atom_site_label
_atom_site_type_symbol
_atom_site_fract_x
_atom_site_fract_y
_atom_site_fract_z
_atom_site_U_iso_or_equiv
_atom_site_adp_type
_atom_site_occupancy
H1     H    0.09050   0.79187  -0.44258   0.00000  Uiso   1.00
H2     H    0.01391   0.93121  -0.42620   0.00000  Uiso   1.00
H3     H    0.05570   0.02766  -0.89013   0.00000  Uiso   1.00
H4     H    0.42787   0.19907  -0.35153   0.00000  Uiso   1.00
H5     H    0.96853   0.79804  -0.74696   0.00000  Uiso   1.00
H6     H    0.52507   0.20293  -0.04334   0.00000  Uiso   1.00
H25    H    0.75000   0.07182  -0.94739   0.00000  Uiso   1.00
H26    H    0.75000   0.86905  -0.52795   0.00000  Uiso   1.00
H27    H    0.75000   0.12054  -0.25699   0.00000  Uiso   1.00
```

N1	N	0.75000	0.15075	-0.05939	0.00000	Uiso	1.00
N2	N	0.75000	0.85412	-0.74253	0.00000	Uiso	1.00
N3	N	0.75000	0.93457	-0.23014	0.00000	Uiso	1.00
H31	H	0.25000	0.11882	-0.05856	0.00000	Uiso	1.00
H32	H	0.25000	0.75207	-0.67504	0.00000	Uiso	1.00
H33	H	0.25000	0.86478	-0.68974	0.00000	Uiso	1.00
H34	H	0.25000	0.01655	-0.65684	0.00000	Uiso	1.00
N7	N	0.25000	0.93547	-0.55621	0.00000	Uiso	1.00
H39	H	0.00000	0.00000	-0.19513	0.00000	Uiso	1.00

```
data_NH5-C2c-100GPa
_audit_creation_date              2014-11-17
_audit_creation_method            'Materials Studio'
_symmetry_space_group_name_H-M    'C2/C'
_symmetry_Int_Tables_number       15
_symmetry_cell_setting            monoclinic
loop_
_symmetry_equiv_pos_as_xyz
  x,y,z
  -x,y,-z+1/2
  -x,-y,-z
  x,-y,z+1/2
  x+1/2,y+1/2,z
  -x+1/2,y+1/2,-z+1/2
  -x+1/2,-y+1/2,-z
  x+1/2,-y+1/2,z+1/2
_cell_length_a                    5.2099
_cell_length_b                    12.2830
_cell_length_c                    4.1377
_cell_angle_alpha                 90.0000
_cell_angle_beta                  89.2438
_cell_angle_gamma                 90.0000
loop_
_atom_site_label
_atom_site_type_symbol
_atom_site_fract_x
_atom_site_fract_y
_atom_site_fract_z
_atom_site_U_iso_or_equiv
_atom_site_adp_type
_atom_site_occupancy
H1    H    1.09353  -0.21647  -0.62320   0.00000  Uiso   1.00
H2    H    0.56676  -0.20548  -0.72984   0.00000  Uiso   1.00
H3    H    0.20886  -0.12371  -0.00252   0.00000  Uiso   1.00
H4    H    1.17046  -0.48017  -0.78604   0.00000  Uiso   1.00
H5    H    0.49731  -0.09118  -0.54024   0.00000  Uiso   1.00
H6    H    1.33833  -0.41657  -0.37796   0.00000  Uiso   1.00
H7    H    1.45920  -0.49600  -0.67765   0.00000  Uiso   1.00
H8    H    1.19157  -0.25999  -0.95282   0.00000  Uiso   1.00
H9    H    1.18169  -0.33098  -0.62074   0.00000  Uiso   1.00
```

N1 N 0.85738 -0.15047 -0.01489 0.00000 Uiso 1.00
N2 N 0.82886 -0.43938 -0.49354 0.00000 Uiso 1.00
H37 H 1.00000 -0.29819 -0.75000 0.00000 Uiso 1.00
H39 H 1.00000 -0.14599 -0.25000 0.00000 Uiso 1.00

data_NH5-C2cII-100GPa

```
_audit_creation_date              2014-11-17
_audit_creation_method            'Materials Studio'
_symmetry_space_group_name_H-M    'C2/C'
_symmetry_Int_Tables_number       15
_symmetry_cell_setting            monoclinic
loop_
_symmetry_equiv_pos_as_xyz
  x,y,z
  -x,y,-z+1/2
  -x,-y,-z
  x,-y,z+1/2
  x+1/2,y+1/2,z
  -x+1/2,y+1/2,-z+1/2
  -x+1/2,-y+1/2,-z
  x+1/2,-y+1/2,z+1/2
_cell_length_a                    4.9791
_cell_length_b                    11.7271
_cell_length_c                    3.9834
_cell_angle_alpha                 90.0000
_cell_angle_beta                  90.6405
_cell_angle_gamma                 90.0000
loop_
_atom_site_label
_atom_site_type_symbol
_atom_site_fract_x
_atom_site_fract_y
_atom_site_fract_z
_atom_site_U_iso_or_equiv
_atom_site_adp_type
_atom_site_occupancy
H1     H     0.10239   0.21829   0.63759   0.00000  Uiso   1.00
H2     H     0.57054   0.20661   0.73995   0.00000  Uiso   1.00
H3     H     0.20014   0.12569   0.99816   0.00000  Uiso   1.00
H4     H     0.66780   0.98069   0.77182   0.00000  Uiso   1.00
H5     H     0.49272   0.09271   0.55011   0.00000  Uiso   1.00
H6     H     0.84269   0.92158   0.37729   0.00000  Uiso   1.00
H7     H     0.95878   0.99430   0.67449   0.00000  Uiso   1.00
H8     H     0.68570   0.76091   0.96206   0.00000  Uiso   1.00
H9     H     0.67429   0.83289   0.62773   0.00000  Uiso   1.00
```

N1 N 0.85554 0.15032 0.00973 0.00000 Uiso 1.00
N2 N 0.32844 0.93875 0.49708 0.00000 Uiso 1.00
H37 H 0.50000 0.80000 0.75000 0.00000 Uiso 1.00
H39 H 0.00000 0.14884 0.25000 0.00000 Uiso 1.00

data_NH5-P2-200GPa

_audit_creation_date 2014-11-17
_audit_creation_method 'Materials Studio'
_symmetry_space_group_name_H-M 'P2'
_symmetry_Int_Tables_number 3
_symmetry_cell_setting monoclinic
loop_
_symmetry_equiv_pos_as_xyz
 x,y,z
 -x,y,-z
_cell_length_a 6.1203
_cell_length_b 3.9144
_cell_length_c 4.7414
_cell_angle_alpha 90.0000
_cell_angle_beta 67.3728
_cell_angle_gamma 90.0000
loop_
_atom_site_label
_atom_site_type_symbol
_atom_site_fract_x
_atom_site_fract_y
_atom_site_fract_z
_atom_site_U_iso_or_equiv
_atom_site_adp_type
_atom_site_occupancy
H1 H 0.45650 0.66168 0.32982 0.00000 Uiso 1.00
H2 H 0.58081 0.77305 0.77049 0.00000 Uiso 1.00
H3 H 0.62393 0.24115 0.34417 0.00000 Uiso 1.00
H4 H 0.40815 0.33033 0.34431 0.00000 Uiso 1.00
H5 H 0.97059 0.73114 0.33690 0.00000 Uiso 1.00
H6 H 0.19814 0.85087 0.36723 0.00000 Uiso 1.00
H7 H 0.12958 0.23245 0.39294 0.00000 Uiso 1.00
H8 H 0.95036 0.35241 0.24853 0.00000 Uiso 1.00
H9 H 0.14364 0.38194 0.98529 0.00000 Uiso 1.00
H10 H 0.71980 0.55872 0.36650 0.00000 Uiso 1.00
H11 H 0.47887 0.99384 0.93629 0.00000 Uiso 1.00
H12 H 0.61761 0.90237 0.50407 0.00000 Uiso 1.00
H13 H 0.87110 0.02814 0.28353 0.00000 Uiso 1.00
H14 H 0.39360 0.18824 0.25631 0.00000 Uiso 1.00
H15 H 0.29845 0.74600 0.89793 0.00000 Uiso 1.00

H16	H	0.57238	0.37565	0.07768	0.00000	Uiso	1.00
H17	H	0.22932	0.52496	0.25038	0.00000	Uiso	1.00
H18	H	0.23104	0.03836	0.05257	0.00000	Uiso	1.00
N1	N	0.70028	0.01152	0.30056	0.00000	Uiso	1.00
N2	N	0.13678	0.52232	0.58733	0.00000	Uiso	1.00
N3	N	0.71750	0.51625	0.98436	0.00000	Uiso	1.00
N4	N	0.87261	0.01924	0.72806	0.00000	Uiso	1.00
H37	H	0.00000	0.68684	-0.00000	0.00000	Uiso	1.00
H38	H	0.00000	0.86552	-0.00000	0.00000	Uiso	1.00
H39	H	0.00000	0.22983	-0.00000	0.00000	Uiso	1.00
H40	H	0.50000	0.49857	0.50000	0.00000	Uiso	1.00

data_NH5-P21c-400GPa
_audit_creation_date 2014-11-17
_audit_creation_method 'Materials Studio'
_symmetry_space_group_name_H-M 'P21/C'
_symmetry_Int_Tables_number 14
_symmetry_cell_setting monoclinic
loop_
_symmetry_equiv_pos_as_xyz
x,y,z
-x,y+1/2,-z+1/2
-x,-y,-z
x,-y+1/2,z+1/2
_cell_length_a 2.5326
_cell_length_b 3.5714
_cell_length_c 4.5278
_cell_angle_alpha 90.0000
_cell_angle_beta 90.8158
_cell_angle_gamma 90.0000
loop_
_atom_site_label
_atom_site_type_symbol
_atom_site_fract_x
_atom_site_fract_y
_atom_site_fract_z
_atom_site_U_iso_or_equiv
_atom_site_adp_type
_atom_site_occupancy
H1 H 0.72418 0.29531 0.98785 0.00000 Uiso 1.00
H2 H 0.20674 0.14193 0.96158 0.00000 Uiso 1.00
H3 H 0.75677 0.48713 0.98242 0.00000 Uiso 1.00
H4 H 0.09027 0.62311 1.23476 0.00000 Uiso 1.00
H5 H 0.47336 0.88792 1.24026 0.00000 Uiso 1.00
N1 N 0.74237 0.87098 0.82676 0.00000 Uiso 1.00
data_NH5-P21c-40GPa
_audit_creation_date 2014-11-17
_audit_creation_method 'Materials Studio'
_symmetry_space_group_name_H-M 'P21/C'
_symmetry_Int_Tables_number 14
_symmetry_cell_setting monoclinic
loop_

```
_symmetry_equiv_pos_as_xyz
  x,y,z
  -x,y+1/2,-z+1/2
  -x,-y,-z
  x,-y+1/2,z+1/2
_cell_length_a                3.3172
_cell_length_b                6.0598
_cell_length_c                5.5053
_cell_angle_alpha             90.0000
_cell_angle_beta              126.9598
_cell_angle_gamma             90.0000
loop_
_atom_site_label
_atom_site_type_symbol
_atom_site_fract_x
_atom_site_fract_y
_atom_site_fract_z
_atom_site_U_iso_or_equiv
_atom_site_adp_type
_atom_site_occupancy
H1    H    0.65971   0.51264   0.39553   0.00000  Uiso   1.00
H2    H    0.79994   0.49683   0.12777   0.00000  Uiso   1.00
H3    H    0.04470   0.58218  -0.11655   0.00000  Uiso   1.00
H4    H    0.72079   0.28471  -0.20080   0.00000  Uiso   1.00
H9    H    1.34358   0.27192   0.43152   0.00000  Uiso   1.00
N1    N    0.63748   0.82091  -0.11550   0.00000  Uiso   1.00
```
